# PyL3dMD: Python LAMMPS 3D molecular descriptors package

**DOI:** 10.1186/s13321-023-00737-5

**Published:** 2023-07-28

**Authors:** Pawan Panwar, Quanpeng Yang, Ashlie Martini

**Affiliations:** Department of Mechanical Engineering, University of California Merced, 5200 North Lake Road, Merced, CA 95343 USA

**Keywords:** LAMMPS, Molecular descriptor, QSPR, Cheminformatics, MD simulations, Python

## Abstract

Molecular descriptors characterize the biological, physical, and chemical properties of molecules and have long been used for understanding molecular interactions and facilitating materials design. Some of the most robust descriptors are derived from geometrical representations of molecules, called 3-dimensional (3D) descriptors. When calculated from molecular dynamics (MD) simulation trajectories, 3D descriptors can also capture the effects of operating conditions such as temperature or pressure. However, extracting 3D descriptors from MD trajectories is non-trivial, which hinders their wide use by researchers developing advanced quantitative-structure–property-relationship models using machine learning. Here, we describe a suite of open-source Python-based post-processing routines, called PyL3dMD, for calculating 3D descriptors from MD simulations. PyL3dMD is compatible with the popular simulation package LAMMPS and enables users to compute more than 2000 3D molecular descriptors from atomic trajectories generated by MD simulations. PyL3dMD is freely available via GitHub and can be easily installed and used as a highly flexible Python package on all major platforms (Windows, Linux, and macOS). A performance benchmark study used descriptors calculated by PyL3dMD to develop a neural network and the results showed that PyL3dMD is fast and efficient in calculating descriptors for large and complex molecular systems with long simulation durations. PyL3dMD facilitates the calculation of 3D molecular descriptors using MD simulations, making it a valuable tool for cheminformatics studies.

**Graphical Abstract:**

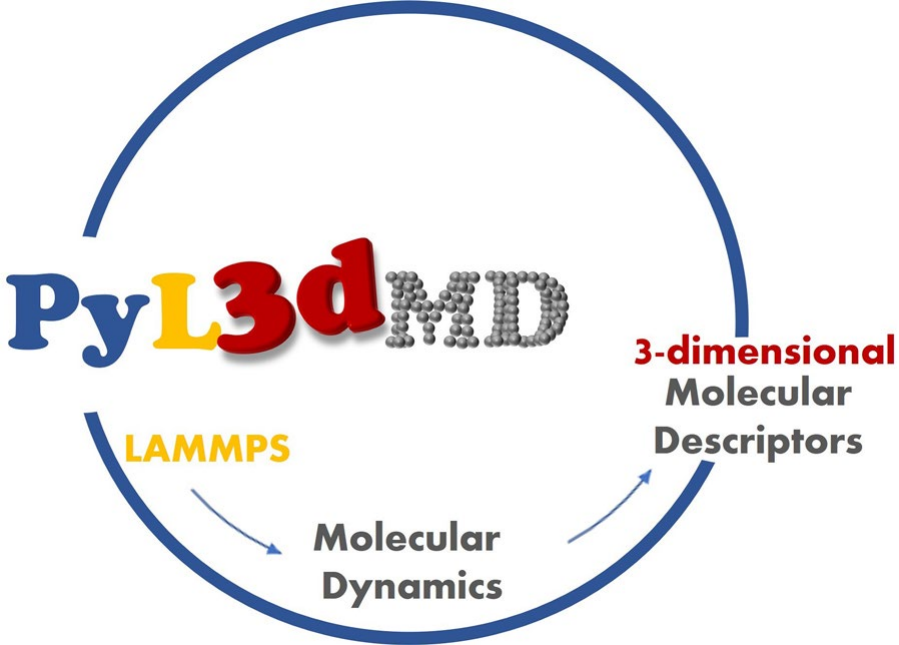

**Supplementary Information:**

The online version contains supplementary material available at 10.1186/s13321-023-00737-5.

## Background

Molecular dynamics (MD) simulations are used to study the physical and chemical properties of materials [1]. There are many software and packages for performing MD simulations, including LAMMPS [2, 3], AMBER [4]Click or tap here to enter text., GROMACS [5], CHARMM [6], Click or tap here to enter text.DESMOND [7], Materials Studio [8], NAMD [9], and QuantumATK [10]. LAMMPS is one of the most widely used open-source packages for MD simulations, attracting particular interest in the scientific research community due to its stability, flexibility, functionality, and responsive community support [1, 2, 11].

Although scientific studies and the development of novel materials have benefited from MD simulations using LAMMPS, the computational cost of atomistic methods still limits the size and time scale of the materials and processes that can be studied. In recent years, with the development of artificial intelligence, significant interest has arisen in machine learning (ML) as a powerful tool for the design and discovery of materials. This approach to predicting material properties is called quantitative-structure–property-relationship (QSPR) modeling and is becoming an essential technology in a wide variety of research fields because of its computational efficiency, scalability, robustness, and predictive ability [12–14].

QSPR modeling is building mathematical relationships between material properties and molecular descriptors of the molecules that compose that material. Molecular descriptors are quantitative representations of physical, chemical, or topological characteristics of molecules that summarize our knowledge and understanding of molecular structure and activity from different aspects [15, 16]. Molecular descriptors play a fundamental role in chemistry, pharmaceutical sciences, environmental protection policy, health research, and quality control. QSPR models based on molecular descriptors have been widely used in pharmaceutical [17, 18] industries and predicting the biological [19] and physiochemical [20–23] of molecules.

There are currently thousands of molecular descriptors, which can be classified into three broad categories: 1D, 2D, and 3D descriptors, where D stands for dimension(s). Simple molecular descriptors derived by counting atom types or structural fragments in the molecule are called constitution or 1D descriptors. Descriptors derived from algorithms applied to a topological representation (molecular graph) are called topological or 2D descriptors. Lastly, there are molecular descriptors derived from geometrical representations of molecules called geometric or 3D descriptors [24]. A descriptor can be simple, like molecular volume, which encodes only one feature of a molecule, or can be complex, like GETAWAY [25], which encodes multiple features—geometry, topology, and atom-weights assembly of a molecule.

Various open-source and proprietary software packages have been developed to calculate descriptors, including PaDEL [26], BlueDesc Dragon [27], RDKit [28], CDK [29], Cinfony [30], Chemopy [31], ChemDes [16], BioJava [32], BioTriangle [33], Bioclipse [34], Propy [35], PyDPI [36], RepDNA [37], CDK-Taverna [38], Protr/ProtrWeb [39], JCompoundMapper [40], ChemmineR [41], and Rcpi [42]. In these packages, a molecular structure must be provided to calculate descriptors for a given molecule. The most common format of input for descriptor calculations is Simplified Molecular-Input Line-Entry System (SMILES) [43].

Although SMILES is easy and fast for calculating simple 1D and 2D descriptors by simple operations such as counting atom types or chemical fragments, it does not contain the information necessary for calculating 3D descriptors, such as the time-dependent geometries of molecules. For calculating 3D descriptors, the available molecular file formats include Sybyl MOL2 files (.mol,.ml2, mol2) by Tripos, Inc., Sybyl Molfiles (.sm2) by ChemOffice, CambridgeSoft Corp., Multiple SD files (.sdf) by Molecular Design Ltd., HyperChem files (.hin) by Hypercube, Inc., MacroModel files by Schrodinger [24]. These files contain geometric information for one molecule and one time instance, so the descriptors are calculated for only a single molecule in a given configuration.

In contrast, MD simulations provide geometric information for multiple molecules and time frames. However, this information cannot be readily used by the currently available packages for calculating 3D descriptors. Specifically, with existing packages, the geometric information for each molecule at each timestep must be converted into the file format required by each package, resulting in the generation of many unnecessary files and computational inefficiency. In addition, most existing packages focus on calculations of simple 1D and 2D descriptors. Finally, available descriptor calculation packages do not directly accept the file and data structures that are output from typical MD simulations, for example, input data files (.lmp) and output trajectory files (.lammpstrj, also called dump files) from LAMMPS. Therefore, there is a need for a tool specifically oriented to 3D descriptors and MD simulations.

Here, this need is addressed by a new Python package, PyL3dMD, where Py stands for Python, L for LAMMPS, 3d for 3-dimensional, and MD for molecular dynamics/descriptors. PyL3dMD in its current form can calculate 2066 3D molecular descriptors (from Refs. [13, 25, 44–79]) directly using the input data and output trajectory files from a LAMMPS simulation.

## General features of PyL3dMD

### Overview of molecular descriptors

PyL3dMD is a robust computational tool capable of calculating more than 2000 3D descriptors [13, 25, 44–79], refer to Table 1 for the reference specific types of molecular descriptors. The currently implemented descriptors are categorized into six sets, as summarized in Table 1. These six sets of descriptors were chosen to be implemented in PyL3dMD because they have been widely utilized in various fields of research, including drug design and discovery, and physicochemical, biological, and pharmacological properties modeling and prediction of *in-silico* molecules and materials [12, 13, 18, 19, 23, 56, 68, 71, 80–84]. The descriptors, which include 3D topology [13, 44, 45], 3D connectivity [45–52], geometric [53–67], GETAWAY [25, 68], CPSA [69, 70], WHIM [71–74], RDF [75], 3D-MoRSE [76, 77], and 3D autocorrelation descriptors (3D Moreau-Broto [78], 3D Moran autocorrelation [78], and 3D Geary autocorrelation [78]), facilitate the prediction of various physicochemical, biological, and pharmacological properties of molecules and materials.

**Table 1:** Summary of the molecular descriptors provided by the current PyL3dMD package, with the number of descriptors within each descriptor set and type

Descriptor set	Descriptor type	Number of descriptors
3D Topological/ Connectivity: set1	• 3D Topology descriptors [13, 44, 45] • 3D Connectivity indices [45–52]	18 9
Geometric: set2	• Dipole moment [53] • Inertia index [13, 54] • Gyration index [13, 55] • Gravitation index [13, 56] • Molecular volume [13, 57] • Shadow indices [13] • Plane of best fit score [58] • Miscellaneous [13, 59–67]	4 16 15 6 2 3 2 41
GETAWAY: set3	• GETAWAY [25, 68]	697
CPSA: set4	• CPSA [69, 70]	30
WHIM: set5	• WHIM [71–74]	112
Miscellaneous: set6	• 3D RDF descriptors [75] • 3D-MoRSE [76, 77] • 3D Moreau-Broto autocorrelation [78] • 3D Moran autocorrelation [78] • 3D Geary autocorrelation [78]	240 240 210 210 210
Property: all	• Density (system property) [79]	1

Density is calculated with all sets of descriptors

3D topology descriptors are used to quantify the connectivity of atoms in three-dimensional space. These descriptors provide information about the bonds between atoms, the topology of the molecular surface, and the shape of the molecule [13, 44, 45]. The 3D connectivity indices are used to describe the interatomic distance between atoms and the angle between bonds [45–52]. Geometric descriptors are used to characterize the shape and size of a molecule [53–67]. GETAWAY (Geometry, Topology, and Atom-Weights Assembly) descriptors are chemical structure descriptors based on the structural and electronic properties of a molecule [25, 68]. CPSA (Charge Polar Surface Area) descriptors are used to quantify the distribution of charge on the surface of a molecule. These descriptors are particularly useful for studying electrostatic interactions and solvation effects [69, 70]. WHIM (Weighted Holistic Invariant Molecular) descriptors are based on the principle of invariance, which means that the descriptors remain the same even if the molecule is transformed or rotated. They are based on statistical indexes calculated by projecting atoms along principal axes. WHIM descriptors capture 3D information regarding molecular size, shape, symmetry, and atom distribution with respect to invariant reference frames [71–74]. 3D radial distribution function (RDF) descriptors are based on the radial distribution function, which describes the probability of finding an atom at a certain distance from another atom. 3D RDF descriptors are based on the radial distribution function and provide information about the spatial distribution of atoms and their environments [75]. 3D-MoRSE (Molecular Surface Electrostatics) stands for 3D-molecule representation of structures based on electron diffraction and these descriptors are based on the electrostatic potentials of the molecular surface [76, 77]. The 3D-MoRSE descriptors translate the 3D coordinates into a molecular code with a modified equation used in electron diffraction studies for preparing theoretical scattering curves [76, 77]. These descriptors provide information about the charge distribution on the surface of a molecule and its three-dimensional shape. Autocorrelation descriptors are based on the autocorrelation of physicochemical properties (charge, mass, van der Waals volume, electronegativity, polarizability, ionization potential, and electron affinity) of atoms within a molecule [78]. These descriptors provide information about the local molecular environment and its correlation with the physicochemical properties of atoms. The complete descriptor list is given in the manual provided in supplementary materials. The governing equations for the descriptors are also provided in the manual. All descriptors currently implemented in PyL3dMD were developed in previous studies [13, 25, 44–79],

### Implementation

PyL3dMD is written in Python, which is readily available and allows for readable code. PyL3dMD uses *argparse* to provide a command-line interface to pass inputs to the package, which allows multiple optional input parameters to be passed efficiently and for automation using shell scripts. PyL3dMD is coded in a module-oriented manner, where each set of descriptors is represented by a module. Furthermore, each module contains a driver function/module used to calculate the respective set of descriptors. This allows PyL3dMD not only to be used through the command line but also to be easily integrated into scripts for user-oriented analyses. Example scripts are provided in the supplementary materials. Owing to the modular structure of PyL3dMD, extensions or new descriptors can be implemented quickly and easily without time-consuming and complex modifications to the source code. To add a new descriptor, users only need to create a new function for that descriptor and call it in the main module. This provides users with the flexibility to either add a new descriptor that is currently unavailable in PyL3dMD or calculate a single descriptor from the existing six descriptor types, specifically for large systems. PyL3dMD has the following dependent Python packages: Math, Pandas, NumPy, Multiprocessing, Time, and Numba, which must be installed before using PyL3dMD. All these packages are by default installed with Anaconda and therefore PyL3dMD does not require any third-party package to be installed. The Numba package, which translates Python functions into optimized machine code at runtime, is used wherever it is possible to significantly increase the computation speed. Numba generates highly optimized machine code that executes much faster than the equivalent pure Python code [85].

An important consideration is the efficiency of the calculation, especially when the system consists of many molecules and trajectories containing data from many time frames. For computational efficiency, PyL3dMD uses matrix algebra wherever possible, rather than for/while loops. PyL3dMD can perform parallel computation for faster estimation of descriptors for all timesteps and molecules. Therefore, it can be used to calculate descriptors for any system size (number of atoms/molecules) irrespective of the complexity of the molecules. If run on desktop computers, PyL3dMD can automatically detect the number of cores available in the system and use them for multiprocessing.

**Fig. 1:**
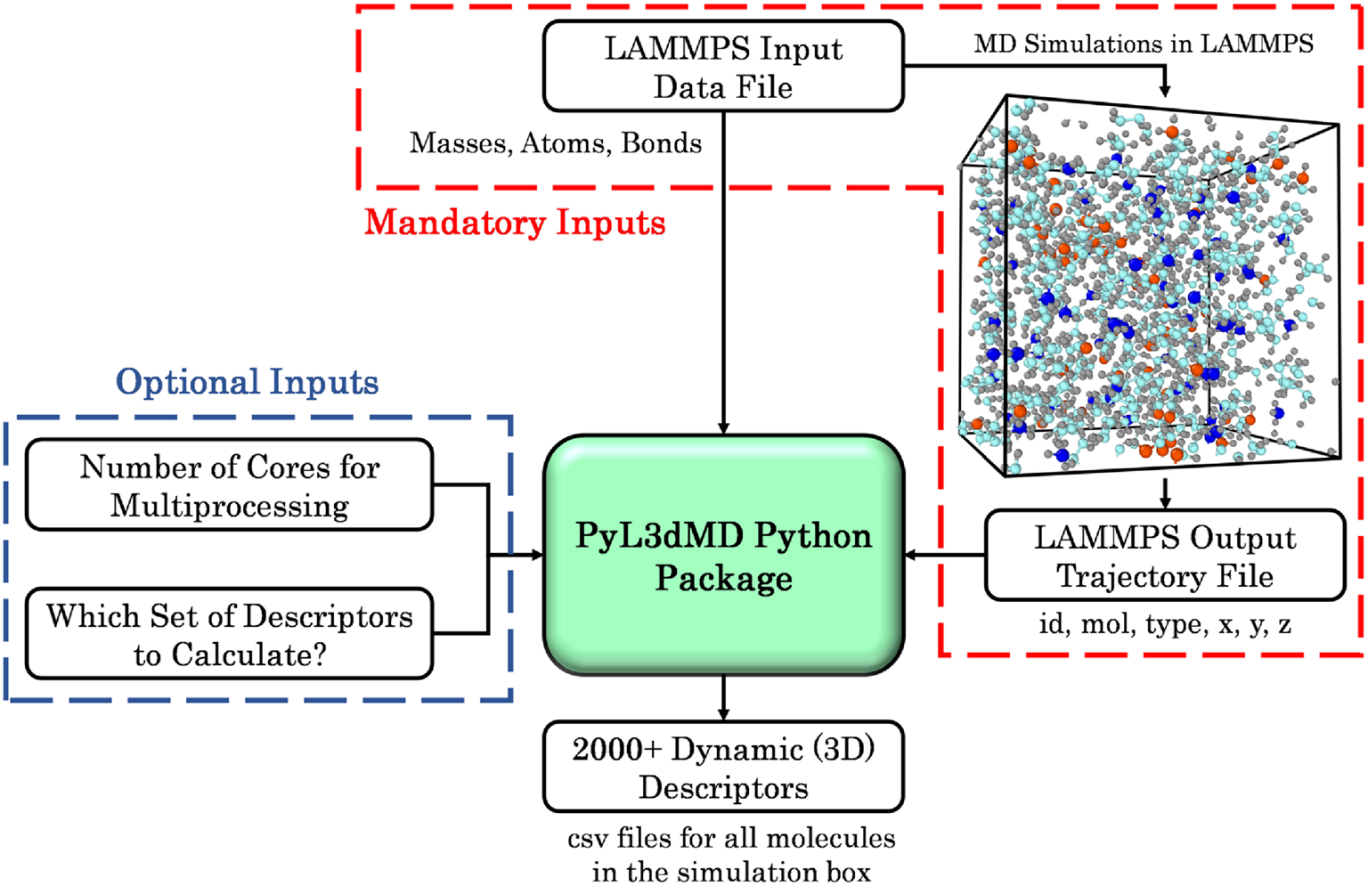
Overview of the PyL3dMD package and its usage

To calculate descriptors, the PyL3dMD package takes four inputs, two of which are mandatory, and two are optional, as shown in Fig. 1. The two mandatory inputs are the LAMMPS input data and output trajectory files, with their file locations. The LAMMPS input data file must have Masses, Atoms, Bonds, Angles, and Dihedrals sections, including charges of the atoms. The LAMMPS trajectory file must have atom id, molecule id, atom type, $x$ (or $xu$), $y$ (or $yu$), and $z$ (or $zu$). Here, $x$, $y$, and $z$ are coordinates of wrapped atoms, whereas $xu$, $yu$, and $zu$ are coordinates of unwrapped atoms. The optional inputs are (1) the integer number of cores (default is maximum available cores/processors) for multiprocessing, and (2) the set number for the descriptors the user would like to calculate (default is all sets of descriptors). Set numbers are defined in Table 1. It is recommended that users provide this optional input argument for faster calculation of molecular descriptors.

On successful execution, PyL3dMD generates a comma-separated values (csv) file for each molecule in the simulation box consisting of molecular descriptors for all time frames. The naming convention for these csv files is molecule_molID.csv, where the molID is the ID of a molecule obtained from the LAMMPS output trajectory file. This way users have freedom to further post-process the calculated descriptors as needed, for example, averaging descriptors over time or multiple molecules.

### Advantages and limitations

PyL3dMD is an intelligent parsing of the LAMMPS input data and output trajectory files. The order of sections (atoms, bonds, angles, dihedrals, etc.) in the LAMMPS input data file can vary. PyL3dMD automatically detects each section to parse relevant information. In addition, LAMMPS allows users to export/write many parameters in any order (that is, in any column) to the output trajectory file. PyL3dMD automatically determines the location of the parameters relevant to the calculation and sorts the coordinates using atom and molecule IDs, which allows users flexibility in the format of the output from LAMMPS.

Another feature of PyL3dMD is that it works with any simulation box size with periodic boundaries, and for any box shape for which all sides of the box are perpendicular to one another, e.g., cubic and orthogonal boxes. LAMMPS allows users to export wrapped or unwrapped $x$, $y$, and $z$ atom coordinates. Although most 3D descriptors are calculated using unwrapped coordinates of atoms, if needed, PyL3dMD automatically converts wrapped coordinates to unwrapped coordinates before any calculations.

Moreover, PyL3dMD can be used with either united-atom (UA) or all-atom (AA) representations. The UA representation combines a group of atoms into a single "united atom" to simplify the simulation; the AA representation, on the other hand, treats each atom in a molecule individually, including hydrogen atoms. The package adds or removes hydrogen atoms depending on the descriptor type. In addition, PyL3dMD can be used for systems with single or multiple molecule types. For instance, PyL3dMD can calculate descriptors for a solution containing both polymer and solvent molecules [21].

Lastly, PyL3dMD itself does not limit the size of the system (number of atoms/molecules) for the descriptor calculation but the maximum size that can be handled efficiently is dependent on the computing resources used.

Regardless of the many advantages, PyL3dMD has several limitations currently.

First, since the PyL3dMD package requires bond, angle, and dihedral information to calculate 3D topological and connectivity descriptors, the package only works for force fields that have this information in the LAMMPS input data file. The package has been tested with the force fields OPLS [86] CVFF [87], and TraPPE-UA [88] but should work for most non-reactive force fields that have Masses, Atoms, Bonds sections in the LAMMPS input data file and have id (ID of atoms), mol (ID of molecules), type (type of atoms), and 3D coordinates ($x$, $y$, $z$) in the LAMMPS output trajectory file. PyL3dMD in its current form does not work for reactive force fields such as ReaxFF [89]. This is because simulations using reactive force fields (e.g., ReaxFF [89]), atom connectivity is not explicitly defined in the data file, but changing over time, this makes it more complicated and computational demanding in descriptor calculations. In the future versions of PyL3dMD, we will overcome this difficulty and incorporate descriptor calculations for simulations with reactive force fields.

Second, PyL3dMD assumes that the atom style is “full” when importing the atom section from the LAMMPS input data file, where “full” is one of the atoms styles for describing the information of each atom in the data file in LAMMPS following the order of “atom ID, molecule ID, atom type, charge, x, y, z”.

Third, PyL3dMD assumes that the LAMMPS simulation is using the “real” unit system (mass = grams/mole, length = Angstroms, time = femtoseconds, energy = kcal/mole, temperature = Kelvin, pressure = atmospheres, electric field = volts/Angstrom, density = gram/cm^3^). However, this may not be an issue for QSPR modeling that is based on the relative values of descriptors for different molecules. The only system property that is calculated is density, so the units of density may need to be converted.

Fourth, PyL3dMD assumes that all the sides of the simulation box are perpendicular to one another when unwrapping the coordinates if the user provides wrapped coordinates. If users provide unwrapped coordinates, PyL3dMD will work for any shape of the simulation box.

Finally, since this package calculates descriptors for each molecule separately over multiple timesteps, it might require a large amount of memory to store arrays, lists, and dictionaries. If a system (simulation box) is very large and the LAMMPS output trajectory file consists of over thousands of timesteps, the package should be run on a high-performance computing (HPC) cluster for faster computations. For such cases, PyL3dMD will work on a regular personal computer but might take longer computational time.

### Example usage

**Fig. 2:**
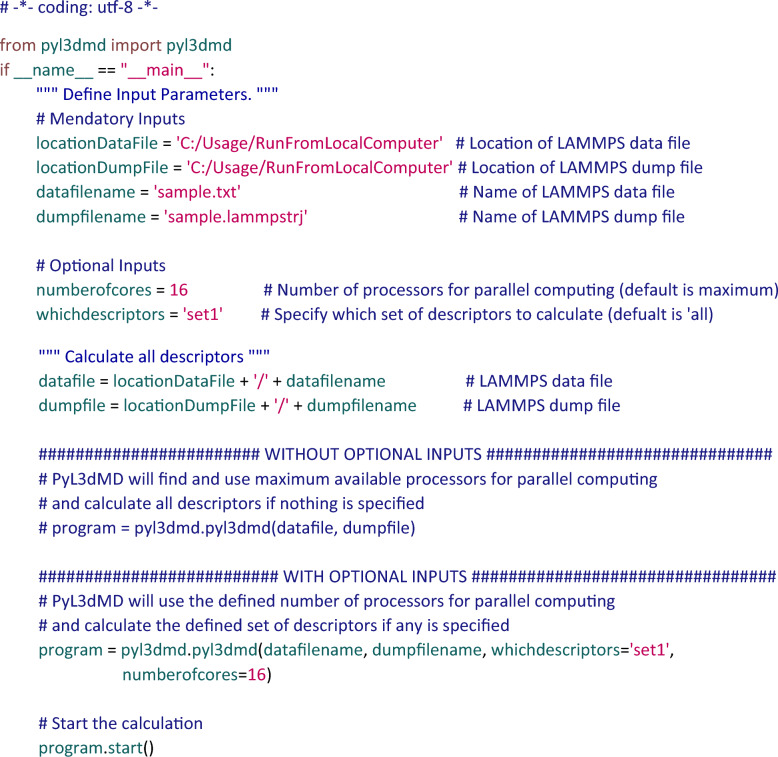
Screenshot of a sample Python script of using PyL3dMD

Fig. 2 shows an example of a Python script that uses the PyL3dMD package for calculating descriptors for a sample model with parallel computations with 16 CPU processors. The sample LAMMPS data file sample.txt and dump file sample.lammpstrj are provided on our GitHub page.

## Application

**Fig. 3:**
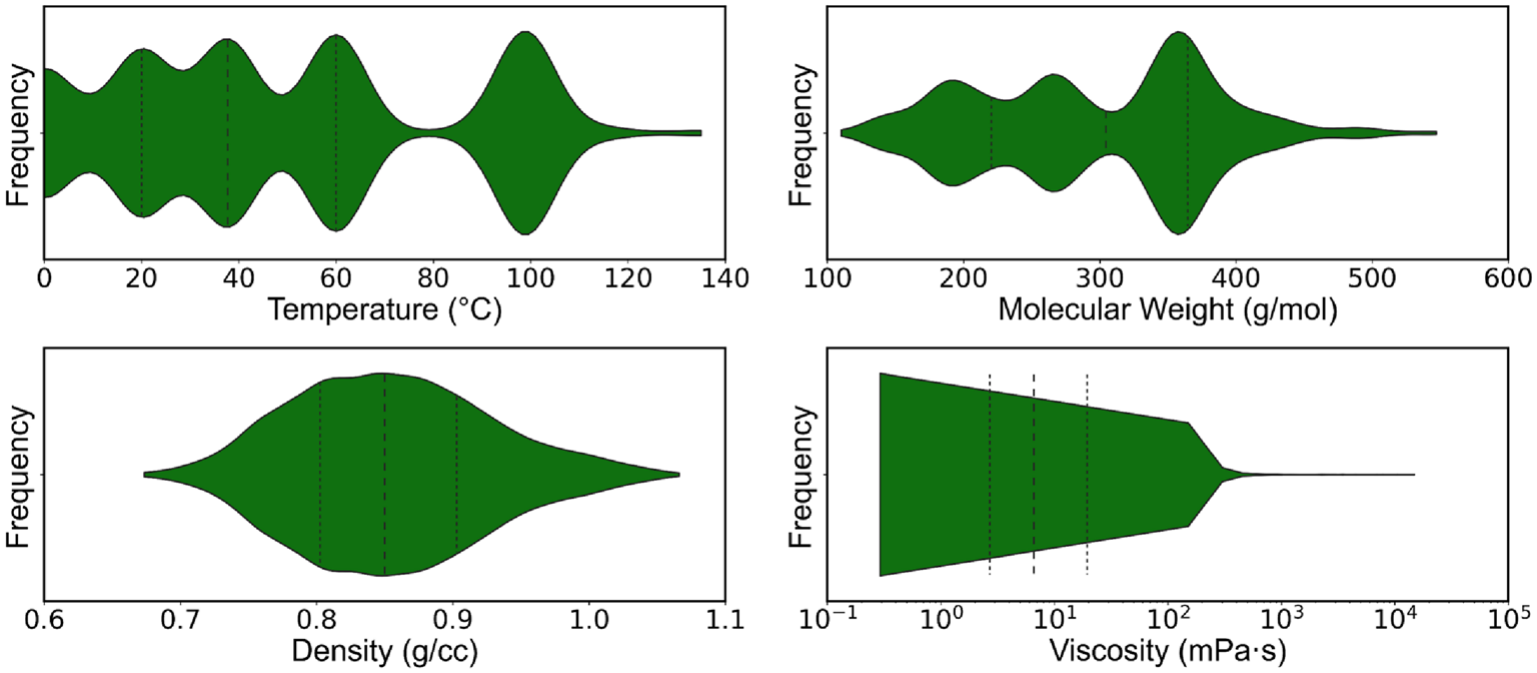
Overview of the experimental data used to generate the MIMO NN. This shows the distributions of temperature, molecular weight, density, and viscosity of the hydrocarbon data used to develop the NN. The dash lines show the quartiles of the distributions

As an example of the application of PyL3dMD, a Multiple-Input Multiple-Output (MIMO) neural network (NN) was developed for predicting temperature-dependent density and viscosity of wide variety of complex hydrocarbons using the molecular descriptors calculated from the PyL3dMD. The experimental dynamic viscosity and density of the hydrocarbons (C_8_ to C_50_) used here were obtained from the American Petroleum Institute (API) Research Project 42 [90] over a wide range of temperatures (0 °C to 135 °C). Figure 3 presents an overview of the experimental data and the distribution of properties. The molecular weights of the hydrocarbons range from 110.2 to 703.3 g/mol, the densities from 0.67 to 1.12 g/cc, and the viscosities from 0.29 to 2.00 × 10^4^ mPa$\cdot$s. This wide range of viscosity for hydrocarbons with C_8_ to C_50_ indicates the complexity in the structure of these hydrocarbons. In total, 1248 data points for 305 hydrocarbons were used to develop the NN. These 1248 data points were randomly divided into three subsets: 70% to train the NN (training dataset), 15% to tune hyperparameters and architecture of the NN (validation dataset), and 15% to assess the performance of the final NN (test dataset) after it has been trained and tuned.

In our previous study [91], which was conducted to compare the predictive capability of molecular descriptors calculated from MD simulations and SMILES code, we conducted MD simulations of these hydrocarbons. The model system (simulation box) for each hydrocarbon consisted of around 5000 atoms (volume of 5.0 nm^3^). The interactions between atoms were described using the OPLS forcefield [86, 92]. Each system was simulated in LAMMPS for 3.0 ns and the trajectories of the atoms in all molecules were stored every 1000 fs. This resulted in a LAMMPS output trajectory file consisting of 3001 time steps. However, only the last 50% timesteps (from the 1500th to the 3001st timesteps) were used to calculate molecular descriptors using PyL3dMD. Then, the descriptors were averaged for developing a MIMO NN.

In our study [91], we also presented a machine learning approach to develop highly predictive models with fewer, simpler, and easily interpretable models. The same machine learning approach was used to select the best molecular descriptors from all the descriptors calculated using PyL3dMD to develop the NN. This approach selected the molecular descriptors getawayHGM, lk, molvolume, and phi4 for further developing NN based on their high correlation with density and viscosity, and less collinearity between these descriptors. Here, getawayHGM is the geometric mean of the leverage magnitude, lk is the Kuhn length, molvolume is the volume of molecule, phi4 is the folding profile 4. Among these, getawayHGM is a GETAWAY descriptor, while lk, molvolume, and phi4 are geometric descriptors. All the calculated molecular descriptors for all hydrocarbons can be accessed from our GitHub page. Readers are referred to Ref. [91] for more details about the MD simulations and machine learning approach.

The model architecture was defined to have multiple dense layers with ReLU activation. It was designed to have multiple input nodes representing the predictor/feature variables and multiple output nodes representing the target variables (density and viscosity). The network was compiled using the Adam optimizer and mean squared error as the loss function for each output, tracking mean absolute error (MSE) as a metric. Then, the network was trained using the training dataset. During the training step, MSE was minimized by adjusting the weights and biases of network. This was achieved through forward propagation and backpropagation, iteratively updating the model parameters using Adam optimizer. Then, using Bayesian optimization, the hyperparameters of NN, including number of neurons, hidden layers, activation functions, learning rate, epoch, and batch size were tuned to improve the model's performance and eliminate the overfitting.

**Fig. 4:**
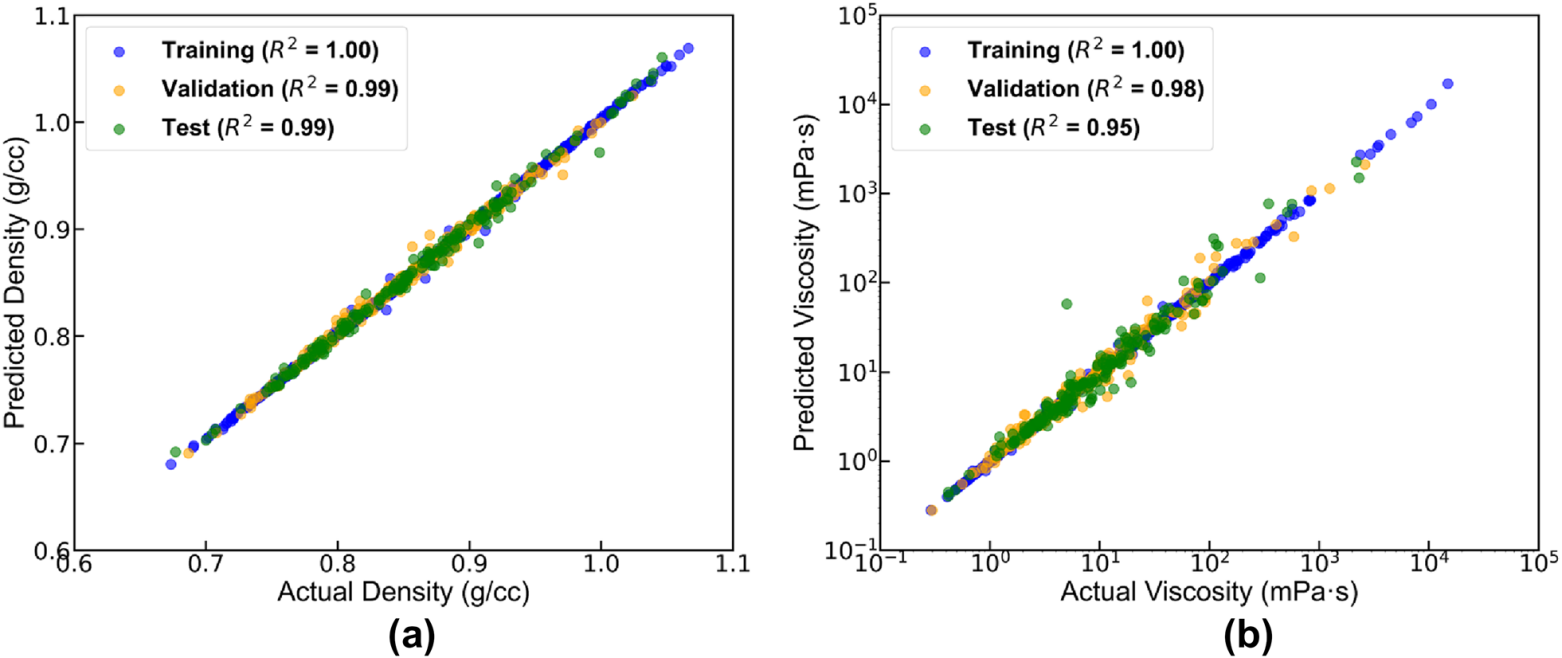
Experimental and model predicted (**a**) density and (**b**) viscosity for the training (blue), validation (orange), and test (green) datasets

Finally, the performance of the final network was evaluated using the R-squared (R^2^) value, calculated for each hydrocarbon property in the training, validation, and test datasets. The Average Variance Inflation Factor (VIF) was also calculated to check for multicollinearity among the input variables. The average VIF for the selected descriptors was 5.2, which suggests that the predictors do not have concerning multicollinearity. Figures 4a, b show the model predicted and actual experimental density and viscosity values over a wide temperature range. The predicted properties for the training, validation, and test datasets are shown as blue, orange, and green circles, respectively. From the statistics in Fig. 4a, b, the NN performed exceptionally well with only four descriptors. Furthermore, the NN was able to predict temperature-dependent properties without incorporating temperature as one of the predictors. This emphasizes the benefit of developing models based on dynamic 3D descriptors calculated from MD simulations, as opposed to static descriptors calculated using SMILES codes.

**Fig. 5:**
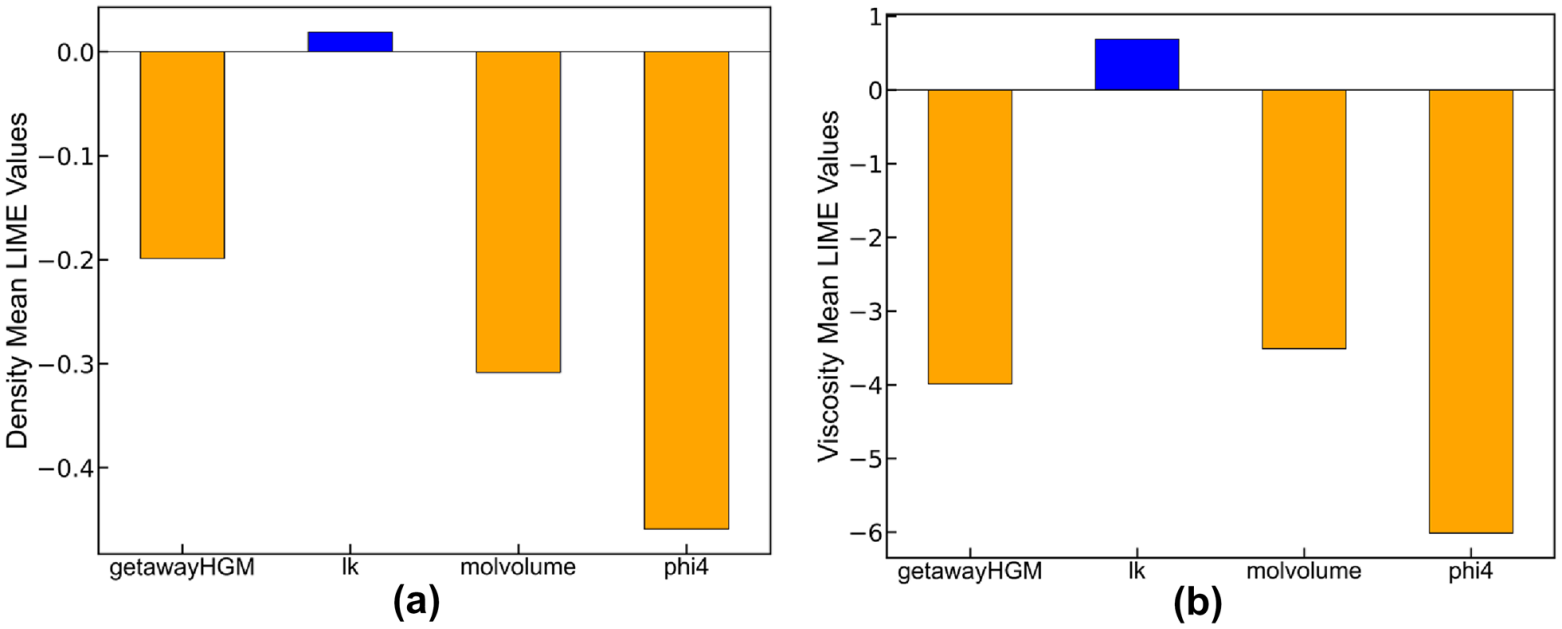
The average LIME value for each feature in neural network for (**a**) density and (**b**) viscosity. The orange and blue colors represent negative and positive relationships, respectively, between a predictor and the response variable. The size of a bar represents the overall importance of a predictor

After evaluating the performance of the developed NN, Local Interpretable Model-Agnostic Explanations (LIME) were utilized to decipher the predictions made by the network. LIME, an ML technique, explains predictions of machine learning based models for individual data points [93]. By computing the mean LIME values of normalized (normalization is very important for side-by-side comparison) features, we got a better understanding of how important each feature is in predicting the density and viscosity of hydrocarbons. These average LIME values, taken from all 1248 data points, are shown in Fig. 5a, b for density and viscosity. This gives us a picture of how each feature influences the output predictions. The higher the LIME value, the more influence a feature has. On the other hand, a lower LIME value means a feature has less influence. The bar charts show that the folding parameter phi4, which quantifies the folding of molecules, had the biggest effect on both density and viscosity predictions. Except for the Kuhn length lk, all other features were negatively correlated to density and viscosity.

## Performance benchmark

**Fig. 6:**
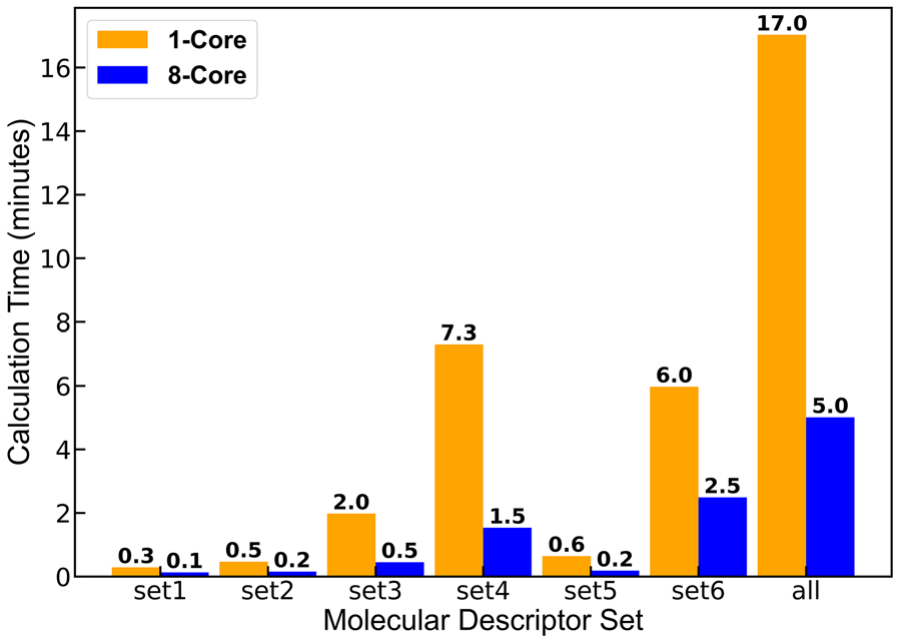
Computation time for each set of descriptors with 1-core (orange bars) and 8-core (blue bars). The number above each bar is the computational time in minutes

The time required for the code to run is a function of the computational resource used (number of cores), system size (number of atoms in the simulation box), and the duration of the MD simulation (number of time frames). The code was tested for a simulation with 10 molecules having 20 atoms per molecule (total 200 atoms in the simulation box) with 1001 timesteps in the LAMMPS output trajectory file. The machine used for this analysis had the following configuration: Intel i7-10700 CPU with 8 cores, 16 processor, 32 GB RAM, and Windows 10 desktop. When multiprocessing with 8 cores was used, PyL3dMD took on average 16 min to calculate all descriptors whereas a single core took 30 min. Figure 6 shows the computational time for each set of descriptors calculated individually and all together. The computation time for CPSA descriptors was considerably higher than the other sets. This analysis demonstrates why it is important to use the optional inputs to eliminate the calculation of unnecessary descriptors. The average time taken to calculate each of these descriptors can be determined by diving the time shown in Fig. 6 by $10 \left(\# molecules\right)\times 1001 \left(\# timesteps\right)=10010$. This results in a very small computation time (~ 0.18 s using a single core and ~ 0.1 s using 8 cores for all descriptors) for a molecule.

Then, the same benchmarking analysis was conducted for a larger simulation box, more realistic size using the same 8 core Windows system. For this benchmarking analysis, the simulation box of around 5000 atoms and LAMMPS output trajectory file of 3001 timesteps was considered which is same as the simulation box size using for 305 hydrocarbons. Windows desktop with 8 cores, PyL3dMD took 6.1, 14.8, 34.2, 139.9, 21.4, 51.0 min in calculating descriptors in the 3D Topological/Connectivity (set1), Geometric (set2), GETAWAY (set3), CPSA (set4), WHIM (set5), and Miscellaneous (set6) sets, respectively.

Since we had to calculate molecular descriptors for 1248 MD simulations conducted at multiple temperatures, we used an HPC cluster with 72 CPU cores to speed up the computation. With the HPC, we were able to calculate molecular descriptors from 1248 large LAMMPS trajectory files (around 5000 atoms and 3001 timesteps) within hours. On average, it took 16.2 min for each simulation file of around 800 megabytes. The hydrocarbon molecules in the simulations had a range of sizes and structures, including n-paraffins, branched-paraffins, 1-olefin, branched-olefins, non-fused ring naphthene, fused ring naphthene, non-fused ring aromatic, and fused ring aromatics, and there was minimal effect of the molecular structure on compute time. This demonstrates the performance of PyL3dMD for large and complex molecular systems and confirms that PyL3dMD is a versatile tool that can be applied to material science studies without scale limitations, while also quantifying the effect of computing resources on computation speed.

**Fig. 7:**
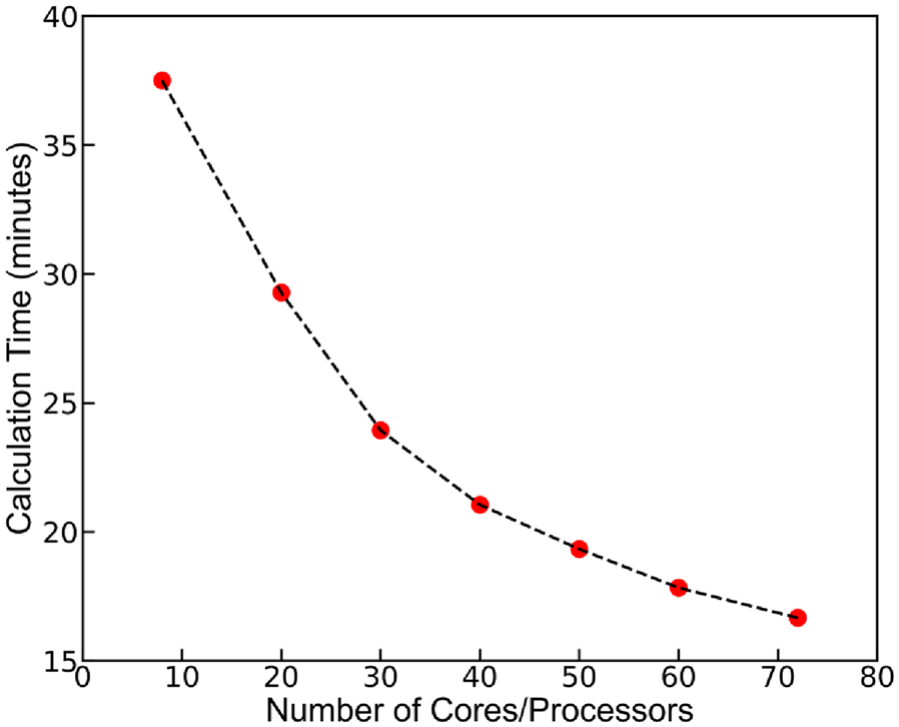
Time taken by PyL3dMD to calculate all molecular descriptors for different number of cores

To benchmark the effect of number of cores/processors on the computational time of all the descriptors, we recorded the calculation time with 8, 20, 30, 40, 50, 60, and 72 processors. This analysis was conducted on the simulation box with around 5000 atoms (more specifically total 4983 atoms and 151 molecules of 33 atoms) and LAMMPS trajectory files of 3001 timesteps. The recorder time for this simulation box with 8, 20, 30, 40, 50, 60, and 72 processors is shown in Fig. 7. This results in Fig. 7 show we can significantly decrease the computation time by increasing the number of cores for processing.

It is important to note that PyL3dMD does not impose any inherent limitations on the scale of the molecular system being analyzed. It is designed to calculate descriptors for simulation data of any size, including data generated from long simulation durations, large simulation boxes, and many molecules within the simulation box. The major factor that affects computation speed is the availability of computing resources for parallel computation. By leveraging parallel computing capabilities, PyL3dMD can effectively handle calculations on a large scale. In the future, we plan to enhance the coding architecture to significantly reduce computation time.

## Request

If you identify any issues or have suggestions for improving PyL3dMD, we welcome you to reach out to us via email or GitHub. We value feedback, questions, and bug reports, and we are open to receiving them through both channels. We also encourage user contributions and invite you to submit a pull request on our GitHub page. This is particularly important as we aim to expand PyL3dMD's capabilities to calculate descriptors for other MD simulation tools.

## Conclusions

Currently, there are several commercial and open-source tools for calculating molecular descriptors, but none of them are compatible with MD simulation tools such as LAMMPS. Here, an open-source Python-based 3D molecular descriptors calculation tool, PyL3dMD, was developed, which is compatible with the formats of LAMMPS input data and output trajectory files. PyL3dMD has been published on GitHub, PyPi, and Conda under the GNU General Public License. PyL3dMD is a multithreaded tool able to utilize multiple CPU cores to increase the efficiency of descriptor calculations. There are two mandatory inputs (LAMMPS input data and output trajectory files) and one optional input (number of cores for multiprocessing) for calculating more than 2000 3D descriptors. PyL3dMD requires minimal user intervention but can also be easily expanded to include more descriptors. The package can be used on all major platforms, including Windows, Linux, and macOS, via Anaconda. In addition, the PyL3dMD package can be easily integrated into custom post-processing scripts. To demonstrate the application of molecular descriptors calculated from PyL3dMD, we developed and presented a multiple-input and multiple-output (MIMO) neural network (NN) to predict density and viscosity of hydrocarbons as functions of temperature. Results and performance benchmark show that PyL3dMD is a versatile tool that can be applied to material science studies with scale only limited by the availability of computing resources.

In the future, PyL3dMD could undergo several improvements to enhance its functionality. These improvements may include increasing its compatibility with various atom styles (e.g., atomic, bond, etc.), expanding the existing descriptor pool, optimizing computational efficiency, ensuring compatibility with other MD simulation packages other than just LAMMPS, and integrating ML algorithms to facilitate QSPR modeling by utilizing the calculated descriptors.

This tool will enable scientific researchers to calculate a wide range of 3D descriptors to quantify molecular chemistry and structure, and ultimately guide the design of advanced materials.

## Supplementary Information

**Additional file 1.** User Manual for PyL3dMD.

## Data Availability

The following supplementary data and materials can be found on GitHub at https://github.com/panwarp/PyL3dMD: Sample script and usage examples for the local computer and HPC. Sample LAMMPS input data and output trajectory files. An excel worksheet with the descriptor of all descriptors. User Manual with installation and usage instructions. Equations used to calculate descriptors are listed in the User Manual. Experimental density and viscosity data, and all the calculated molecular descriptors of 305 hydrocarbons.
