# Supplementary material for: PyL3dMD: Python LAMMPS 3D molecular descriptors package
Source: J Cheminform. 2023 Jul 28;15:69. doi: 10.1186/s13321-023-00737-5 (PMC10385924; doi:10.1186/s13321-023-00737-5)
Supplement: Supplementary file 1 — Additional file 1. User Manual for PyL3dMD. [file 13321_2023_737_MOESM1_ESM.pdf]

User Manual for PyL3dMD

Python LAMMPS 3D Molecular Descriptors Package

Pawan Panwar[1*], Quanpeng Yang[1], and Ashlie Martini[1+]

[1]Department of Mechanical Engineering, University of California Merced,
5200 North Lake Road, Merced, CA 95343, USA

*E-mail: ppanwar@ucmerced.edu (panwarp@msoe.edu)

+E-mail: amartini@ucmerced.edu

Table of Content

1. Introduction 2
2. Installation Instructions 4
3. Example Usage of the Package 5
4. Governing Equations 9
   4.1. Property Descriptors 9
   4.2. Topology and Connectivity Descriptors 9
   4.3. Geometric Descriptors 12
   4.4. Weighting Schemes 21
   4.5. GETAWAY Descriptors 21
   4.6. WHIM Descriptors 24
   4.7. RDF Descriptors 25
   4.8. Autocorrelation Descriptors 25
   4.9. 3D-MoRSE Descriptors 26
Bibliography 27

1. Introduction

Molecular descriptors characterize the biological, physical, and chemical properties of molecules and have long been used for understanding molecular interactions and facilitating materials design. Some of the most robust descriptors are derived from geometrical representations of molecules, called geometric or 3-dimensional (3D) descriptors. When calculated from molecular dynamics (MD) simulation trajectories, 3D descriptors can also capture the effects of operating conditions such as temperature or pressure. However, extracting 3D descriptors from MD trajectories is non-trivial, which hinders their wide use by researchers developing advanced quantitative-structure-property-relationship models using machine learning. Here, we describe a suite of open-source Python-based post-processing routines, called PyL3dMD, for calculating 3D descriptors from MD simulations. PyL3dMD is compatible with the popular simulation package LAMMPS and enables users to compute more than 2000 3D molecular descriptors from atomic trajectories generated by MD simulations.

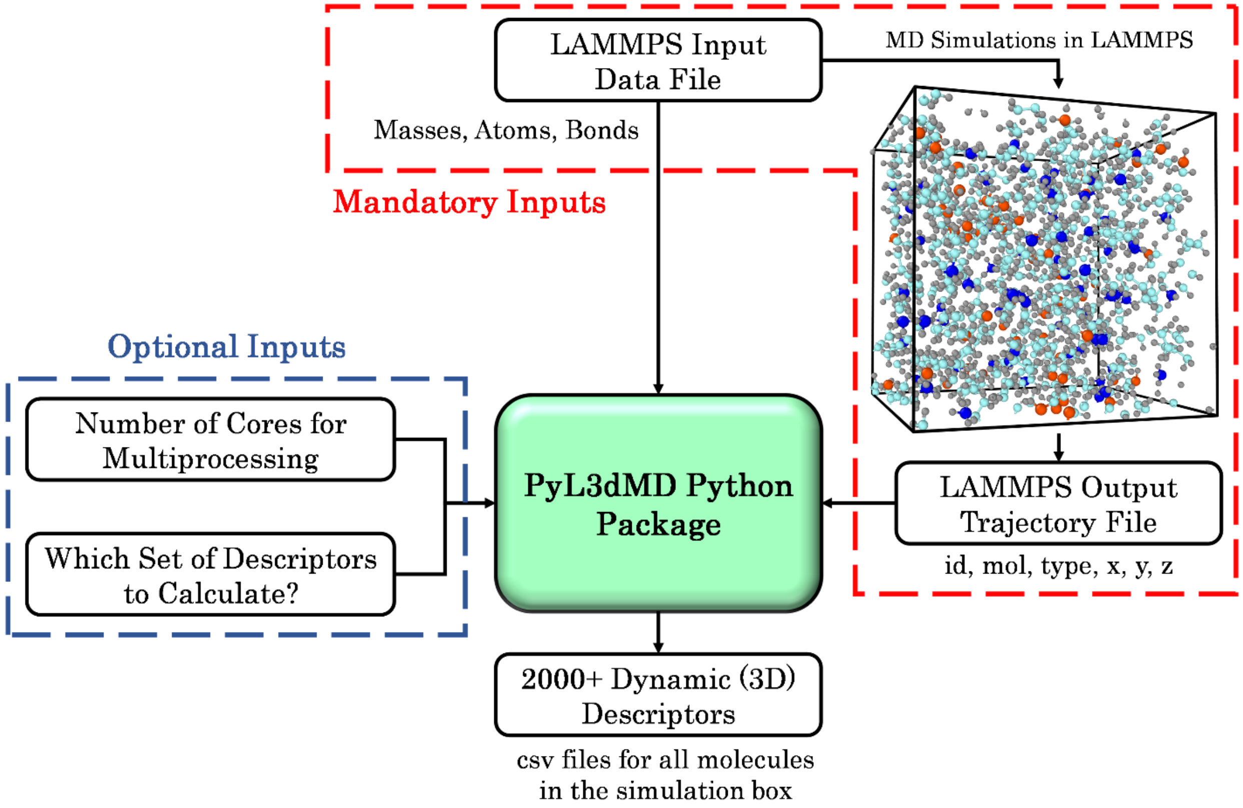

Figure 1: Overview of the PyL3dMD package and its usage.

Table 1: Summary of the molecular descriptors provided by the current PyL3dMD package, with the number of descriptors within each descriptor set and type. Density is calculated with all set of descriptors.

Descriptor Set	Descriptor Type	Number of Descriptors
3D Topological/ Connectivity: `set1`	• 3D Topology descriptors<br>• 3D Connectivity indices	18<br>9
Geometric: `set2`	• Dipole moment<br>• Inertia index<br>• Gyration index<br>• Gravitation index<br>• Molecular volume<br>• Shadow indices<br>• Plan of best fit score<br>• Miscellaneous	4<br>16<br>15<br>6<br>2<br>3<br>2<br>41
GETAWAY: `set3`	• GETAWAY	697
CPSA: `set4`	• CPSA	30
WHIM: `set5`	• WHIM	112
Miscellaneous: `set6`	• 3D RDF descriptors (RDF)<br>• 3D-MoRSE (MoRSE)<br>• 3D Moreau-Broto autocorrelation (ATS)<br>• 3D Moran autocorrelation (MATS)<br>• 3D Geary autocorrelation (GATS)	240<br>240<br>210<br>210<br>210
Property: `all`	• Density (system property)	1

The detailed list of descriptors with their description can be found in the excel worksheet on the GitHub: https://github.com/panwarp/PyL3dMD.

2. Installation Instructions

For simplification purposes, users should use Anaconda (https:// www.anaconda.com) to install Python and packages.

Install on a Windows or iOS system:

1. From here, download the Anaconda for the Windows or iOS operating system.
2. Install Anaconda by double clicking on the Anaconda installer "`.exe`" file and following the prompt on the installer.
3. The package has been uploaded to PyPi (https://pypi.org/project/PyL3dMD/). Users can install the package using the command "`pip install pyl3dmd`".
    i. Run "`Anaconda Prompt`" as the administrator.
    ii. Copy and paste "`pip install pyl3dmd`" to "Anaconda Prompt".
    iii. Press "`Enter`" on the keyboard to start the installation.
    iv. The installation will finish in seconds.

Install on the Linux Platform: Since the PyL3dMD package supports parallel computing using multiple CPUs, users can accelerate the descriptor calculation using a high-performance computer (HPC) cluster. The installation on an HPC is similar:

1. From here, download the Anaconda for the Linux operating system.
2. Log into the cluster using any terminal software, e.g., Putty.
3. Copy and upload the downloaded bash (`.sh` file) installer file to the cluster using software, e.g., WinScP.
4. Run the installer script with bash by typing bash followed by the name of the bash installer file:
   ```
   bash Anaconda3-5.2.0-Linux-x86_64.sh
   ```
5. source the `.bashrc` file to add Anaconda to your PATH by typing:
   ```
   cd ~

   source .bashrc
   ```
   Now that Anaconda3 is installed and Anaconda3 is added to our PATH, source the `.bashrc` file to load the new PATH environment variable into the current terminal session. Note the `.bashrc` file is in the home directory. You can see it with `ls -a`.
6. To verify the installation is complete, open Python from the command line by typing:
   ```
   python
   ```
   If you see Python from Anaconda listed, your installation is complete. Exit the Python REPL, by typing: `exit()`
7. Install package: "`pip install pyl3dmd`".
8. The installation will finish in seconds.

Note: To avoid any error, please install the most recent version of NumPy.

To uninstall PyL3dMD, users can use command "`pip uninstall pyl3dmd`".

3. Example Usage of the Package

To make getting started easier, we provided some sample files. These files contain the usage of the package on a local computer and an HPC found on GitHub page at https://github.com/panwarp/PyL3dMD.

Here is the structure of the files, where there are two folders (one for local computer and the other for HPC):

```
¦---- RunFromLocalComputer
¦           sample.lmp
¦           sample.lammpstrj
¦           sample.py

¦---- RunFromHPC
¦           sample.lmp
¦           sample.lammpstrj
¦           sample.py
¦           submit.sh
```

For running the package on a local computer, files in the "`RunFromLocalComputer`" can be used, where there are a LAMMPS data file (`sample.lmp`), a LAMMPS output trajectory file (`sample.lammpstrj`), and a Python file (`sample.py`).

Here is the content of the `sample.py` file the local computer:

```
# -*- coding: utf-8 -*-

from pyl3dmd import pyl3dmd
if __name__ == "__main__":
    """
    Define Input Parameters
    """
    # Mendatory Inputs
    locationDataFile = 'C:/Usage/RunFromLocalComputer' # Location of your LAMMPS data file
    locationDumpFile = 'C:/Usage/RunFromLocalComputer' # Location of your LAMMPS dump file
    datafilename = 'sample.txt' # Name of your LAMMPS data file
    dumpfilename = 'sample.lammpstrj' # Name of your LAMMPS dump file

    # Optional Inputs
    numberofcores = 16 # Number of processors for parallel computing (default is maximum)
    whichdescriptors = 'set1' # Specify which set of descriptor to calculate (default is all')

    """
    Calculate all descriptors
    """
    datafile = locationDataFile + '/' + datafilename # Your LAMMPS data file
    dumpfile = locationDumpFile + '/' + dumpfilename # Your LAMMPS dump file

    ######################### WITHOUT OPTIONAL INPUTS #################################
    # PyL3dMD will find and use maximum available processors for parallel computing
    # and also calculate all descriptors if nothing is specified
    # program = pyl3dmd.pyl3dmd(datafile, dumpfile)

    ########################### WITH OPTIONAL INPUTS ###################################
    # PyL3dMD will use the defined number of processors for parallel computing
    # and also calculate the defined set of descriptors if any is specified
    program = pyl3dmd.pyl3dmd(datafilename, dumpfilename, whichdescriptors='set1', numberofcores=16)

    # Start the calculation
    program.start()
```

This Python file is ready to be run to calculate the '`set1`' descriptors based on the provided data and trajectory files using 16 CPU processors for parallel computation. However, if users wish to calculate descriptors for their own models, the names and paths of their own data and trajectory files should be provided in `sample.py`. Besides, which descriptors to be calculated and the number of CPU processors can be customed by users. Generally, more processors will accelerate the calculation. If optional inputs are not provided, PyL3dMD will calculate all the descriptors using the maximum available processors in a system.

After running this Python code, multiple `.csv` files (one file for each molecule) containing the descriptors of the model will be generated.

To run the package on an HPC, here are the steps to follow:

1. Upload the files in the “RunFromHPC” folder to the HPC
2. Use the command "`sbatch submit.sh`" to submit the job to the HPC

Here is the content of the `sample.py` file for the HPC:

```
# -*- coding: utf-8 -*-

from pyl3dmd import pyl3dmd
if __name__ == "__main__":
    """
    Define Input Parameters
    """
    # Mendatory Inputs
    locationDataFile = 'C:/Usage/RunFromLocalComputer' # Location of your LAMMPS data file
    locationDumpFile = 'C:/Usage/RunFromLocalComputer' # Location of your LAMMPS dump file
    datafilename = 'sample.txt' # Name of your LAMMPS data file
    dumpfilename = 'sample.lammpstrj' # Name of your LAMMPS dump file

    # Optional Inputs
    numberofcores = 16 # Number of processors for parallel computing (default is maximum)
    whichdescriptors = 'set1' # Specify which set of descriptor to calculate (default is all')

    """
    Calculate all descriptors
    """
    datafile = locationDataFile + '/' + datafilename # Your LAMMPS data file
    dumpfile = locationDumpFile + '/' + dumpfilename # Your LAMMPS dump file

    ######################### WITHOUT OPTIONAL INPUTS #################################
    # PyL3dMD will find and use maximum available processors for parallel computing
    # and also calculate all descriptors if nothing is specified
    program = pyl3dmd.pyl3dmd(datafile, dumpfile)

    ########################### WITH OPTIONAL INPUTS ###################################
    # PyL3dMD will use the defined number of processors for parallel computing
    # and also calculate the defined set of descriptors if any is specified
    # program = pyl3dmd.pyl3dmd(datafilename, dumpfilename, whichdescriptors='set1', numberofcores=16)

    # Start the calculation
    program.start()
```

Here is the content of the `submit.sh` file for the HPC:

```
#!/bin/bash
#SBATCH --job-name=PyL3dMD
#SBATCH --partition=desktop
#SBATCH --nodes=1
#SBATCH --cpus-per-task=72
#SBATCH --time=168:00:00
#SBATCH --export=ALL
#SBATCH --mail-type=ALL
#SBATCH --mail-user=panwarp@msoe.edu
echo $SLURM_JOB_NODELIST > nodelist.out
echo "Slurm gave us $SLURM_CPUS_ON_NODE CPU(S) on this node."
module purge
python sample.py
```

4. Governing Equations

The equation of the descriptors is listed in the following section [1–9].

4.1. Property Descriptors

Density - The simulation-calculated density is calculated by the ratio of total mass ($mass$) of atoms in the simulation box and the volume ($volume$) of simulation box at an operating condition:

$$\rho = \frac{mass}{volume} \tag{1}$$

4.2. Topology and Connectivity Descriptors

Adjacency Matrix — The adjacency matrix $\mathbf{A}$ is a square symmetric of size $n_A \times n_A$, where n_A is the number of vertices (atoms). The entries a_{ij} of the adjacency matrix $\mathbf{A}$ are equal one if vertices v_i and v_j are adjacent (i.e., the atoms i and j are bonded), and zero otherwise. Mathematically [1,3],

$$[\mathbf{A}]_{ij} = \begin{cases} 1 & \text{if } (i,j) \in \mathrm{E(G)} \\ 0 & \text{otherwise} \end{cases} \tag{2}$$

Where $\mathrm{E(G)}$ is the set of graph edges (bonds). The adjacency matrix is usually derived from the H-depleted molecular graph.

Distance matrix — The distance matrix $\mathbf{D}$ is a square symmetric of size $n_A \times n_A$, where n_A is the number of vertices (atoms). It is sometimes also called 2D-distance matrix or topological distance matrix. The entries d_{ij} of the matrix $\mathbf{D}$ are the topological distances between all the pairs of graph vertices. The topological distance d_{ij} is the number of edges (bonds) along the shortest path ${}^{\min}P_{ij}$ between the vertices v_i and v_j. The diagonal entries the distance matrix equal zero whereas the off-diagonal entries equal one if vertices v_i and v_j are adjacent (that is, the atoms i and j are bonded and $d_{ij} = a_{ij} = 1$, where a_{ij} are elements of the adjacency matrix **A**) and are greater than one otherwise. Mathematically [1,3],

$$[\mathbf{D}]_{ij} = \begin{cases} d_{ij} = |{}^{\min}P_{ij}| & \text{if } i \neq j \\ 0 & \text{if } i = j \end{cases} \tag{3}$$

The adjacency matrix $\mathbf{A}$ encodes information about vertex connectivity and the distance matrix $\mathbf{D}$ encodes information about relative locations of graph vertices [1,3].

Reciprocal Distance Matrix - The reciprocal distance matrix $\mathbf{D}^{-1}$ is a square symmetric of size $n_A \times n_A$, where n_A is the number of vertices (atoms). The entries of reciprocal distance matrix are the inverse of entries d_{ij} of the topical distance matrix $\mathbf{D}$. Mathematically [1,3],

$$[\mathbf{D}^{-1}]_{ij} = \begin{cases} d_{ij}^{-1} & \text{if } i \neq j \\ 0 & \text{if } i = j \end{cases} \tag{4}$$

Vertex Degree Matrix — The vertex degree matrix **V** is a square symmetric of size $n_A \times n_A$, where n_A is the number of vertices (atoms), whose diagonal entries are the vertex degrees δ_i. Mathematically [1,3],

$$[\mathbf{V}]_{ij} = \begin{cases} \delta_i \text{ if } i = j \\ 0 \ \ \text{if } i \neq j \end{cases} \tag{5}$$

Molecular Matrix — The molecular matrix **M** is a rectangular matrix of size $n_A \times 3$ where n_A is the number of vertices (atoms). The entries of **M** are the atom spatial coordinates (x, y, z) of the molecule. Mathematically [1,3],

$$\mathbf{M} = \begin{bmatrix} x_1 & y_1 & z_1 \\ x_2 & y_2 & z_2 \\ \vdots & \vdots & \vdots \\ x_{n_A} & y_{n_A} & z_{n_A} \end{bmatrix}$$

Geometrical Distance Matrix — The geometrical distance matrix (**G**) is a square symmetric of size $n_A \times n_A$, where n_A is the number of vertices (atoms). It is sometimes called 3D-distance matrix or Euclidean distance matrix. Each entry r_{ij} of the geometrical distance matrix is the Euclidean distance (intramolecular interatomic distance) between the atoms i and j, that is, between each pair of atoms of a molecule. Mathematically [1,3],

$$\mathbf{G} = \begin{vmatrix} 0 & r_{12} & \cdots & r_{1A} \\ r_{21} & 0 & \cdots & r_{2A} \\ \cdots & \cdots & \cdots & \cdots \\ r_{A1} & r_{A2} & \cdots & 0 \end{vmatrix} \quad \text{or} \quad [\mathbf{G}]_{ij} = \begin{cases} r_{ij} & \text{if } i \neq j \\ 0 & \text{if } i = j \end{cases} \tag{6}$$

The geometry matrix does not contain information about atom connectivity. Therefore, it is accompanied by a connectivity table (list of all bonded atoms).

Reciprocal Geometry Matrix — The reciprocal geometry matrix $\mathbf{G}^{-1}$ is obtained by inverting the Euclidean distance collected in the geometry matrix, as [1,3]:

$$[\mathbf{G}^{-1}]_{ij} = \begin{cases} r_{ij}^{-1} \text{ if } i \neq j \\ 0 \quad \text{if } i = j \end{cases} \tag{7}$$

3D-Adjacency Matrix — The 3D-adjacency matrix ($\mathbf{A}^{\mathrm{G}}$) is obtained from the geometry matrix as [1,3]:

$$\mathbf{A}^{\mathrm{G}} = \mathbf{G} \otimes \mathbf{A} \tag{8}$$

where $\otimes$ indicates the Hadamard matrix product (or pairwise multiplication) and **A** is the adjacency matrix. Thus, the elements of the 3D-adjacency matrix are the bond lengths for pairs of bonded atoms, and zero otherwise [1,3].

Reciprocal squared geometrical distance matrix — The Reciprocal squared geometrical distance matrix (**RG**) is a square symmetric matrix containing the inverse of the squared Euclidean distances, taken pairwise, between the atoms of a molecule. Mathematically [1,3],

$$[\mathbf{RG}]_{ij} = \begin{cases} r_{ij}^{-2} \text{ if } i \neq j \\ 0 \quad \text{if } i = j \end{cases}$$

Distance/distance matrix — The Distance/distance matrix ($\mathbf{G/D}$) is a square and symmetric matrix containing the ratios between the Euclidean distance and the topological distance, between each pair of atoms of a molecule. Mathematically [1,3],

$$[\mathbf{G/D}]_{ij} \text{ or } [\mathbf{DD}]_{ij} = \begin{cases} \dfrac{r_{ij}}{d_{ij}} & \text{if } i \neq j \\ 0 & \text{if } i = j \end{cases}$$

The equation of topology and connectivity descriptors are listed in the following section [1,3]:

Geometric distance degree:

$$G_{\sigma_i} = \sum_{j=1}^{A} r_{ij} \tag{9}$$

Average geometric distance degree:

$$G_{\bar{\sigma}} = \frac{1}{A} \cdot \sum_{i=1}^{A} G_{\sigma_i} = \frac{1}{A} \cdot \sum_{i=1}^{A} \sum_{j=1}^{A} r_{ij} \tag{10}$$

3D-Wiener index:

$$3D_{W_H} = \frac{1}{2} \cdot \sum_{i=1}^{A} \sum_{j=1}^{A} r_{ij} \tag{11}$$

3D-connectivity indexes:

$$m_{XX_t} = \sum_{k=1}^{K} \left(\prod_{i=1}^{n} G_{\sigma_i} \right)_k^{-1/2} \tag{12}$$

Euclidean connectivity index:

$$X^E = \sum_{i=1}^{A-1} \sum_{j=i+1}^{A} \left(G_{\sigma_i} \cdot G_{\sigma_j} \right)^{-1/2} \tag{13}$$

3D-Balaban index:

$$3D_J = \frac{B}{C+1} \cdot \sum_{i=1}^{A-1} \sum_{j=i+1}^{A} a_{ij} \cdot \left(G_{\sigma_i} \cdot G_{\sigma_j} \right)^{-1/2} \tag{14}$$

Variant of the geometric distance degree:

$$3DW_i = \sum_{j=1}^{A} \left(1 - a_{ij}\right) \cdot \exp\left(r_{ij}^{-2}\right) \tag{15}$$

Zagreb-like 3DM_1:

$$3DM_1 = \sum_{i=1}^{A} 3DW_i \tag{16}$$

Zagreb-like 3DM_2:

$$3DM_2 = \sum_{i=1}^{A-1} \sum_{j=i+1}^{A} a_{ij} \cdot \left(3DW_i \cdot 3DW_j\right) \quad (17)$$

Connectivity type $3D^0X$:

$$3D^0X = \sum_{i=1}^{A} (3DW_i)^{-1/2} \quad (18)$$

Connectivity type $3D^1X$:

$$3D^1X = \sum_{i=1}^{A-1} \sum_{j=i+1}^{A} a_{ij} \cdot \left(3DW_i \cdot 3DW_j\right)^{-1/2} \quad (19)$$

Wiener-type indexes (3D Wi):

$$3DWi = \sum_{i=1}^{A-1} \sum_{j=i+1}^{A} \exp\left(r_{ij}\right) \quad (20)$$

Geometric eccentricity:

$$G_{\eta_i} = \max_j\left(r_{ij}\right) \quad (21)$$

Geometric radius:

$$G_R = \min_i\left(G_{\eta_i}\right) \quad (22)$$

Geometric diameter:

$$G_D = \max_i\left(G_{\eta_i}\right) \quad (23)$$

Geometrical shape coefficient:

$$I_3 = \frac{G_D - G_R}{G_R} \quad (24)$$

3D-Schultz index:

$$3D_{\mathrm{MTI}} = \sum_{i=1}^{A} [(b_{\mathbf{A}} + \mathbf{G}) \cdot \mathrm{v}]_i \quad (25)$$

3D-MTI' index:

$$3D_{MTI'} = \sum_{i=1}^{A} \sum_{j=1}^{A} |\mathbf{A}.\mathbf{G}|_{ij} = \sum_{i=1}^{A} [\mathbf{v}^{\mathrm{T}} \cdot \mathbf{G}]_i \quad (26)$$

4.3. Geometric Descriptors

The equation of geometric descriptors is listed in the following section [1,3]:

Molar Volume - The molar volume is defined as the ratio of the volume of a sample of that substance (expressed in liters, for example) to the amount of substance (usually expressed in moles) in the sample. It is experimentally measured as:

$$\bar{V} = \frac{M_w}{\rho} \quad (27)$$

Here, M_w is the molecular weight and ρ is the density of the liquid. The SI unit of molar volume is cubic meters per mole (m^3/mol).

Molecular Volume - The molecular volume V is defined as the volume of the region within a molecule is constrained by its neighbors. It is calculated from density ρ of the liquid as:

$$V = \frac{M_w}{\rho . N_A} = \frac{\bar{V}}{N_A} \tag{28}$$

Geometric Center — The geometric center r_{gc} of a molecule is the arithmetic mean of position of the atoms in a molecule [1,3].

$$r_{gc} = \frac{1}{n_A}\sum_{i=1}^{n_A} r_i \tag{29}$$

It is calculated by averaging value of atom coordinates separately for each axis as:

$$x_{gc} = \frac{1}{n_A}\sum_{i=1}^{n_A} x_i \qquad y_{gc} = \frac{1}{n_A}\sum_{i=1}^{n_A} y_i \qquad z_{gc} = \frac{1}{n_A}\sum_{i=1}^{n_A} z_i \tag{30}$$

Variance — The distribution of atoms with respect to its center can be calculated by the variance as [1,3]:

$$s^2 = \frac{\sum_{i=1}^{n_A}\left(r_i - r_{gc}\right)^2}{n_A - 1} \tag{31}$$

Here, r_i is the position of atom. The square root of the variance is called the standard deviation (s).

Pearson's first index — A measure of the asymmetry of the distribution of atoms can be calculated as [1,3]:

$$k_3 = \frac{\sum_{i=1}^{n_A}\left(r_i - r_{gc}\right)^3}{s^3 n_A} \tag{32}$$

where s^3 the third power of its standard deviation. If $k_3 < 0$ then a right-tailed distribution, and if $k_3 > 0$, then a left-tailed distribution.

Kurtosis - A measure of the degree of bimodality of the distribution of atoms can be calculated as [1,3]:

$$k_4 = \frac{\sum_{i=1}^{n_A}\left(r_i - r_{gc}\right)^4}{s^4 n_A} \tag{33}$$

where s^4 the fourth power of its standard deviation. $k = \infty$ for a peak distribution, $k = 1$ for a complete bimodal distribution, $k = 1.8$ for uniform distributions, and $k = 3$ for normal distribution.

Center of Mass — The center of mass r_{cm} of a molecule is the arithmetic mean of mass-position of the atoms in a molecule. The center of mass r_{cm} of the molecule is calculated with the following formula [10,11].

$$r_{cm} = \frac{1}{M_w}\sum_{i=1}^{n_A} m_i r_i \tag{34}$$

It is calculated separately for each axis as:

$$x_{cm} = \frac{1}{M_w}\sum_{i=1}^{n_A} m_i x_i \qquad y_{cm} = \frac{1}{M_w}\sum_{i=1}^{n_A} m_i y_i \qquad z_{cm} = \frac{1}{M_w}\sum_{i=1}^{n_A} m_i z_i \tag{35}$$

Here, m_i is the atomic mass of ith atom and $M_w = \sum_{i=1}^{n_A} m_i$.

End-to-end Distance — A simple size descriptor known as end-to-end distance of a molecule is defined as [10–12]:

$$R_{ee} = \|\vec{r}_{1\text{st}} - \vec{r}_{\text{last}}\|$$

$$R_{ee} = \sqrt{(x_{1\text{st}} - x_{\text{last}})^2 + (y_{1\text{st}} - y_{\text{last}})^2 + (z_{1\text{st}} - z_{\text{last}})^2} \tag{36}$$

Here, R_{ee} is the interatomic distance between the first and the last atoms of the chain and $\vec{r}$ is the vector of the atom coordinates with respect to the center of mass.

Radius of Gyration — The radius of gyration R_g is used to describe the dimensions of a molecule, more specifically, the distribution of atomic masses in a molecule. Therefore, it is a measure of molecular compactness. A small value is obtained when most of the atoms are close to the center of mass. The radius of gyration of a molecule is defined as [10,11],

$$R_g = \sqrt{\frac{1}{M_w}\sum_{i=1}^{n_A} m_i (r_i - r_{cm})^2} = \sqrt{\frac{1}{M_w}\sum_{i=1}^{n_A} m_i [(x_i - x_{cm})^2 + (y_i - y_{cm})^2 + (z_i - z_{cm})^2]} \tag{37}$$

Here, $M_w = \sum_{i=1}^{n_A} m_i$ and r_{cm}, respectively, are the total mass and center-of-mass position of the molecule and m_i and r_i, respectively, are mass and position of ith atom.

Gravitational Indices — Geometrical descriptors reflecting the mass distribution in a molecule, defined as [1,3]:

$$G_1 = \sum_{i=1}^{n_A - 1} \sum_{j=i+1}^{n_A} \frac{m_i m_j}{r_{ij}^2} \tag{38}$$

$$G_2 = \sum_{b=1}^{n_B} \left(\frac{m_i m_j}{r_{ij}^2}\right)_b \tag{39}$$

where m_i and m_j are the atomic masses of the considered atoms, r_{ij}, the corresponding interatomic distances, and A and B the number of atoms and bonds of the molecule, respectively. The G_1 index considers all atom pairs in the molecule while the G_2 index is restricted to pairs of bonded atoms. These indices are related to the bulk cohesiveness of the molecules, accounting, simultaneously, for both atomic masses (volumes) and their distribution within the molecular space. For modelling purposes, the square root and cube root of the gravitational indices were also proposed [1,3].

Mean Square Radius of Gyration — The mean square radius of gyration for the unperturbed chains can be derived from the Lagrange theorem:

$$S^2 = \frac{1}{2n_A^2}\sum_{i=1}^{n_A}\sum_{j=1}^{n_A} r_{ij}^2 \tag{40}$$

Hydrodynamic Radius — The hydrodynamic radius R_h of a molecule is obtained from the mean reciprocal distance:

$$\frac{1}{R_h} = \frac{1}{2n_A^2}\sum_{i=1}^{n_A}\sum_{j=1}^{n_A}\frac{1}{r_{ij}} \tag{41}$$

Radius of Gyration Tensor - The gyration tensor of the conformations is defined as [10,11],

$$\mathbf{GY} = \frac{1}{M}\begin{bmatrix} \sum_{i=1}^{n_A} m_i(x_i - x_{cm})^2 & \sum_{i=1}^{n_A} m_i(x_i - x_{cm})(y_i - y_{cm}) & \sum_{i=1}^{n_A} m_i(x_i - x_{cm})(z_i - z_{cm}) \\ \sum_{i=1}^{n_A} m_i(x_i - x_{cm})(y_i - y_{cm}) & \sum_{i=1}^{n_A} m_i(y_i - y_{cm})^2 & \sum_{i=1}^{n_A} m_i(y_i - y_{cm})(z_i - z_{cm}) \\ \sum_{i=1}^{n_A} m_i(x_i - x_{cm})(z_i - z_{cm}) & \sum_{i=1}^{n_A} m_i(y_i - y_{cm})(z_i - z_{cm}) & \sum_{i=1}^{n_A} m_i(z_i - z_{cm})^2 \end{bmatrix} \tag{42}$$

Eigenvalues of Gyration Tensor and Shape Parameters

Since the gyration tensor S is a symmetric 3x3 matrix, a Cartesian coordinate system can be found in which it is diagonal (eigen) [10,11],

$$\mathbf{GY} = \begin{bmatrix} \lambda_x & 0 & 0 \\ 0 & \lambda_y & 0 \\ 0 & 0 & \lambda_z \end{bmatrix} \tag{43}$$

Here, the axes are chosen such that the diagonal elements are ordered $\lambda_x \leq \lambda_y \leq \lambda_z$. These diagonal elements are called the principal moments of the gyration tensor [10,11]. The squared radius of gyration is the sum of the principal moments, i.e., $R_g^2 = \lambda_x + \lambda_y + \lambda_z$ [10,11].

Normalized Principal Gyration Ratios — Normalization can be performed by dividing the two lower eigenvalues of gyration tensor (λ_1 and λ_2) by the highest value (λ_3), generating two characteristic values of normalized principal gyration ratios (NGRs) for a molecule (λ_{N1} and λ_{N2}) as:

$$\lambda_{N1} = \frac{\lambda_1}{\lambda_3} \tag{44}$$

$$\lambda_{N2} = \frac{\lambda_2}{\lambda_3} \tag{45}$$

The three parameters that describe the shape can determined by the principal moments of the gyration tensor as [10,11],

Acylindricity — The acylindricity is always non-negative and zero only when the two principal moments are equal. The condition, $c = 0$ is met when the distribution of particles is cylindrically symmetric which can also be true when the particle distribution is symmetric with respect to the two coordinate axes, e.g., when the particles are distributed uniformly on a regular prism.

$$c = \lambda_z - 0.5(\lambda_y + \lambda_x) \tag{46}$$

Asphericity — It measures the deviation from spherical symmetry. The asphericity is always non-negative and zero only when the three principal moments are equal. The condition, $b = 0$ is met when the distribution of particles is spherically symmetric which can also be true when the particles are distributed uniformly on a cube, tetrahedron or other Platonic solid.

$$b = \lambda_y - \lambda_x \tag{47}$$

Relative shape anisotropy — It reflects both the symmetry and dimensionality of a polymer conformation. This parameter is limited between the values of 0 (if all points are spherically symmetric) and 1 (if all points lie on a line). It reaches 1 for an ideal linear chain and drops to zero for highly symmetric conformations. For planar symmetric objects, the relative shape anisotropy converges to the value of 1/4 [11].

$$k = \frac{3}{2}\frac{\lambda_x^2 + \lambda_y^2 + \lambda_z^2}{\left(\lambda_x + \lambda_y + \lambda_z\right)^2} - \frac{1}{2} \tag{48}$$

Inertia tensor - These are physical quantities related to the rotational dynamics of a molecule. The moment of inertia about any axis is defined as [1]:

$$\mathbf{I} = \sum_{i=1}^{n_A} m_i {r_i}^2$$

Here, n_A is the atom number, and m_i and r_i are the atomic mass and the perpendicular distance from the chosen axis of the ith atom of the molecule, respectively. For any rectangular coordinate system, the inertia tensor can be given as [1]

$$\mathbf{I} = \begin{bmatrix} \sum_{i=1}^{n_A} m_i({y_i}^2 + {z_i}^2) & \sum_{i=1}^{n_A} m_i x_i y_i & \sum_{i=1}^{n_A} m_i x_i z_i \\ \sum_{i=1}^{n_A} m_i x_i y_i & \sum_{i=1}^{n_A} m_i({x_i}^2 + {z_i}^2) & \sum_{i=1}^{n_A} m_i y_i z_i \\ \sum_{i=1}^{n_A} m_i x_i z_i & \sum_{i=1}^{n_A} m_i y_i z_i & \sum_{i=1}^{n_A} m_i({x_i}^2 + {y_i}^2) \end{bmatrix} \tag{49}$$

The unit of gyration tensor is distance² where the unit of inertia tensor is mass×distance². Although they have different units, the gyration tensor is related to the moment of inertia tensor. The key difference is that the particle positions are weighted by mass in the inertia tensor, whereas the gyration tensor depends only on the particle positions; mass plays no role in defining the gyration tensor [1].

Eigenvalues of Inertia Tensor and Shape Parameters [1,3]

The eigenvalues of inertia tensor are called the principal moments of inertia. Principal moments of inertia are the moments of inertia corresponding to that particular and unique orientation of the axes for which one of the three moments has a maximum value, another a minimum value, and the third is either equal to one or the other or is intermediate in value between the other two. The corresponding axes are called principal axes of a molecule (or principal inertia axes). Moreover, the products of inertia all reduce to zero and the corresponding inertia matrix is diagonal. Conventionally, principal moments of inertia are labeled as: $I_A \leq I_B \leq I_C$.

In general, the three principal moments of inertia have different values, but, depending on the molecular symmetry, they show characteristic equalities such as those shown in Table 2.

Table 2: Type of symmetry for some common case of principal moment [1,3].

**Principal Moments**	**Type of Symmetry**
$I_A = I_B = I_C$	Spherical top
$I_A = I_B \neq I_C$	Symmetric top
$I_A \neq I_B \neq I_C$	Asymmetric top
$0 = I_A \neq I_B = I_C$	Linear
$I_A + I_B = I_C$	Planar

Normalized Principal Moment Inertia Ratios — Normalization can be performed by dividing the two lower principal moments of inertia values (I_1 and I_2) by the highest value (I_3), generating two characteristic values of normalized principal moments of inertia ratios (NPRs) for a molecule (I_{N1} and I_{N2}) as [13]:

$$I_{N1} = \frac{I_1}{I_3} \tag{50}$$

$$I_{N2} = \frac{I_2}{I_3} \tag{51}$$

Normalization eliminates the dependency of the chosen representation on the size of the molecules under investigation. Furthermore, due to the intrinsic characteristics of the inertia tensor, the following relation will be fulfilled:

$$\frac{I_2}{I_3} \geq max\left(\frac{I_1}{I_3}, 1 - \frac{I_1}{I_3}\right)$$

$[I_1/I_3, I_2/I_3]$ equals [1,1], [0.5,0.5], and [0,1], corresponding to archetype “envelope” shapes of spheres, disks, and rods, respectively.

Inertial shape factor — This is a shape factor based on the principal moments of inertia and defined as [1,3]:

$$S_I = \frac{I_2}{I_1 \cdot I_3} \quad (52)$$

Here, I_1, I_2, and I_3 are the principal moments of inertia].

Molecular eccentricity - It is a shape descriptor obtained from the eigenvalues I_i of the inertia matrix defined as [1,3]:

$$\varepsilon = \frac{(I_3^2 - I_1^2)^{1/2}}{I_3} \quad (53)$$

where $\varepsilon = 0$ corresponds to spherical top molecules and $\varepsilon = 1$ to linear and planar molecules. It is a shape descriptor defined by analogy with the eccentricity of an ellipse, which is defined as [1,3]:

$$\varepsilon = \frac{(l_M^2 - l_m^2)^{1/2}}{l_M}; 0 \leq \varepsilon \leq 1 \quad (54)$$

where l_M and l_m are the lengths of the major and minor elliptical axes, respectively.

Asphericity — A descriptor that measures the deviation from the spherical shape [1,3], calculated from the eigenvalues I_i of the inertia matrix as [1,3]:

$$\Omega_A = \frac{1}{2}\frac{(I_3 - I_2)^2 + (I_3 - I_1)^2 + (I_2 - I_1)^2}{I_1^2 + I_2^2 + I_3^2}; 0 \leq \Omega_A \leq 1 \quad (55)$$

where $\Omega_A = 0$ corresponds to spherical top molecules and $\Omega_A = 1$ to linear molecules. For prolate molecules (cigar shaped), $I_3 > I_2 \approx I_1$ and $\Omega_A \approx 1$, whereas for oblate molecules (disk shaped) $I_3 \approx I_2 > I_1$ and $\Omega_A \approx 0.5$.

Spherosity index — An anisometry descriptor defined as a function of the eigenvalues [1,3]:

$$\Omega_S = \frac{3I_1}{I_1 + I_2 + I_3}; 0 \leq \Omega_S \leq 1 \quad (56)$$

Spherosity index varies from zero for flat molecules, such as benzene, to one for totally spherical molecules [1,3].

Linearity index — Based on a similar approach of the unweighted WHIM shape index K_u, it is defined as [1,3]:

$$L_i = \sqrt{\frac{I_3/I_2/I_1}{M_w^2}} \times 100 \quad (57)$$

where the inertia moments are calculated on the unweighted atom coordinates and M_w is the molecular weight. High values are associated with small linear molecules (4–7), whereas small values are associated with highly branched nonlinear molecules (0–0.5).

Characteristic ratio — A descriptor of average shape features of polymer. It can be considered as a measure of the degree of folding, defined as [1,3]:

$$C_{\infty} = \lim_{B\to\infty} C = \frac{\langle R_G^2\rangle}{B.\,l^2} \quad (58)$$

Here, $\langle R_G^2\rangle$ is the mean square radius of gyration averaged on all the conformations (or configurations), B the number of bonds, and l is the Kuhn length.

The characteristic ratio is also defined for the end-to-end distance r_{ee}, as [1,3]:

$$C'_{\infty} = \lim_{B\to\infty} C' = \frac{\langle r_{ee}^2\rangle}{B.\,l^2} \quad (59)$$

Span

A size descriptor defined as the radius of the smallest sphere, centered on the center of mass, completely enclosing all atoms of a molecule [1,3]:

$$R = \max_i(r_i) \quad (60)$$

Here, r_i is the distance of the ith atom from the center of mass. The average span descriptor, calculated as the average value of conformational changes and denoted by $\bar{R}$ is used to describe long chain molecules and is related to the Kuhn length.

Kuhn length - For long chain molecules, the Kuhn length is the mean of the bond distances, i.e.,

$$l = \frac{\sum_{b=1}^{n_B}\left(r_{ij}\right)_b}{n_B} \quad (61)$$

where B is the number of bonds and r_{ij} is the bond distance between i and j bonded atoms [12].

Contour Length — The contour length L_C is defined as:

$$L_C = n_B.\,l \quad (62)$$

Size-shape geometrical constant - It is derived from the radius of gyration as [Wilding and Rowley, 1986]:

$$\alpha_G = -7.706\times10^{-4} + 0.033R_G + 0.01506R_G^2 - 9.997\times10^{-4}R_G^3 \quad (63)$$

Plane of Best Fit Score - The average distance of all heavy atoms from the plane of best fit is proposed as a descriptor known as plane of best fit (PBF) score [14]. The coordinates of heavy atoms in the molecule are fitted to the plan with normalized equation $Ax + By + Cz + D = 0$.

The equation of best fit is then used to give the distance, Δ, of each heavy atom from the plane as

$$\Delta = \frac{|Ax_i + By_i + Cz_i + D|}{\sqrt{A^2 + B^2 + C^2}} \quad (64)$$

$$\text{PBF Score} = \frac{1}{n_A}\sum_{i=1}^{n_A}\Delta_i \quad (65)$$

$$\text{Normalized PBF Score} = \frac{1}{n_A}\frac{1}{n_A}\sum_{i=1}^{n_A}\Delta_i \quad (66)$$

The plane of best fit (PBF) score, has a theoretical range of [0, ∞). However, in practice, the PBF score tends to be below two for small drug-like molecules and below ten for proteins [14]. By normalizing the PBF score to the number of heavy atoms, it is applicable to any molecule irrespective of molecular size. The PBF score separates molecules closely clustered in NPR space, thereby allowing greater granularity of 3D shape characterization in molecular design and compound selection [14].

Molecular descriptors for distance/distance matrix [1,3]:

Molecular profiles:

$$\{1_D, 2_D, 3_D, 4_D, 5_D, 6_D, \ldots\} \quad (67)$$

Here,

$$k_D = \frac{1}{k!}\frac{\sum_{i=1}^{A}\sum_{j=1}^{A} r_{ij}^k}{A} \quad (68)$$

Average distance/distance degree:

$$ADDD = \frac{1}{A}\cdot\sum_{i=1}^{A}\sum_{j=1}^{A}\frac{r_{ij}}{d_{ij}} \quad j \neq i \quad (69)$$

D/D index:

$$D/D = \frac{1}{2}\cdot\sum_{i=1}^{A}\sum_{j=1}^{A}\frac{r_{ij}}{d_{ij}} \quad j \neq i \quad (70)$$

Folding degree index:

$$\emptyset = \frac{\lambda_1^{DD}}{A} \quad 0 < \emptyset < 1 \quad (71)$$

Folding profile:

$$\{1_\emptyset, 2_\emptyset, 3_\emptyset, \ldots, k_\emptyset, \ldots\} \quad (72)$$

***g*-factor** — It is a measure of branching which is calculated by the ratio of S^2 for a given branched chain to that for the linear chain with the same molecular weight. It is also equal to the Wiener index normalized by the Wiener index of the linear chain.

$$g = \frac{S^2}{(S^2)_{\text{linear}}} = \frac{W}{W_{linear}} \quad (73)$$

Here, the Wiener index of linear chains $W_{linear} = \frac{1}{6}n_A(n_A^2 - 1)$

4.4. Weighting Schemes

The following atomic properties are used as the atomic weightings for molecular descriptor calculation in PyL3dMD are:

- unitary weight (u)
- atomic charge (c)
- atomic mass (m)
- van der Waals volume (V)
- atom electronegativity (E_n)
- atom polarizability (α_a)
- ionization potential (I_P)
- electron affinity (E_A)

Atomic properties are typically used in carbon-scaled form. That is, the ratio between the atomic property and the atom property of carbon. The atomic properties used in the PyL3dMD are directly taken from the ChemoPy [2]package.

Atomic properties are not always defined for the whole periodic table. Molecular descriptors based on a property that is not available for all atoms of a molecule will result in 'na'. Therefore, for united atom potential, all the weighted molecular descriptors will result in 'na'.

This weighing scheme is applied to GETAWAY, WHIM, RDF, ATS, MATS, GATS, and MoRSE descriptors.

4.5. GETAWAY Descriptors

ETAWAY stands for Geometry, topology, and atom-weights assembly descriptors that have been proposed as chemical structure descriptors derived from the molecular influence matrix $\mathbf{H}$, its diagonal elements h_{ii} called **leverages**, and influence/distance matrix $\mathbf{R}$. Leverages encode information related to the "influence" of each atom of a molecule in determining the whole shape of a molecule [1,3].

Molecular Influence Matrix — The molecular influence matrix $\mathbf{H}$ is a square symmetric of size $n_A \times n_A$, where n_A is the number of vertices (atoms). It shows rotational invariance with respect to the molecule coordinates. It is calculated as [1]:

$$\mathbf{H} = \mathbf{M} \cdot (\mathbf{M}^T \cdot \mathbf{M})^{-1} \cdot \mathbf{M}^T \tag{74}$$

where $\mathbf{M}$ is the molecular matrix comprising the centered cartesian coordinates x, y, z of the molecule atoms, including hydrogen, in a chosen conformation. Atomic coordinates are assumed to be calculated with respect to the geometrical center of the molecule to obtain translational invariance.

Influence/Distance Matrix – The influence/distance matrix $\mathbf{R}$ is a square symmetric of size $n_A \times n_A$, where n_A is the number of vertices (atoms). It encodes spatial relationships between pairs of atoms of a molecule. The diagonal elements of the matrix $\mathbf{R}$ are zero, while each off-diagonal element is calculated by the ratio of the geometric mean of the corresponding ith

and jth diagonal elements of the matrix $\mathbf{H}$ over the interatomic distance r_{ij} provided by the geometry matrix $\mathbf{G}$. Mathematically [1,3],

$$[\mathbf{R}]_{ij} = \begin{cases} \sqrt{h_i \cdot h_j}/r_{ij} & \text{if } i \neq j \\ 0 & \text{if } i = j \end{cases} \tag{75}$$

Here, h_i and h_j are the leverages of the atoms i and j, and r_{ij} is their geometric distance. The square root product of the leverages of two atoms is divided by their interatomic distance to make less significant contributions from pairs of atoms far apart, according to the basic idea that interactions between atoms in the molecule decrease as their distance increases. Obviously, the largest values of the matrix elements are derived from the most external atoms (i.e., those with high leverages) and simultaneously next to each other in molecular space (i.e., those having small interatomic distances) [3].

Most of the GETAWAY descriptors are simply calculated only by the leverages used as the atomic weightings [1,3].

Geometric mean of the leverage magnitude:

$$H_{GM} = 100 \cdot \left(\prod_{i=1}^{n_A} h_{ii} \right)^{1/n_A} \tag{76}$$

Total information content on the leverage equality:

$$I_{TH} = A_0 \cdot \log_2 A_0 - \sum_{g=1}^{G} N_g \cdot \log_2 N_g \tag{77}$$

Standardized information content on the leverage equality

$$I_{SH} = \frac{I_{TH}}{A_0 \cdot \log_2 A_0} = 1 - \frac{\sum_{g=1}^{G} N_g \cdot \log_2 N_g}{A_0 \cdot \log_2 A_0} \tag{78}$$

Mean information content on the leverage magnitude:

$$HIC = -\sum_{i=1}^{An_A} \frac{h_{ii}}{M} \cdot \log_2 \frac{h_{ii}}{M} \tag{79}$$

Average row sum of the influence/distance matrix:

$$RARS = \frac{1}{n_A} . \sum_{i=1}^{n_A} \sum_{j=1}^{n_A} \frac{\sqrt{h_{ii} \cdot h_{jj}}}{r_{ij}} = \frac{1}{n_A} \cdot \sum_{i=1}^{n_A} VS_i(\mathbf{R}) \tag{80}$$

R-connectivity index:

$$RCON = \sum_{i=1}^{n_A} \sum_{j=1}^{n_A} a_{ij} \cdot \left(VS_i(\mathbf{R}) \cdot VS_j(\mathbf{R}) \right)^{1/2} \tag{81}$$

R-matrix leading eigenvalue:

$$REIG = S_pMax(\mathbf{R}) \tag{82}$$

GETAWAY descriptors based on autocorrelation functions – The GETAWAY descriptors also comprised of autocorrelation vectors obtained by double-weighting the molecule atoms in such a way as to account for various atomic properties with 3D information encoded by the elements of the molecular influence matrix **H** and influence/distance matrix **R** [1,3].

HATS indexes:

$$HATS_k(w) = \sum_{i=1}^{n_A-1} \sum\nolimits_{j>i} (w_i \cdot h_{ii}) \cdot (w_j \cdot h_{jj}) \cdot \delta(d_{ij}; k) \quad k = 0,1,2,\dots,D \tag{83}$$

HATS total index:

$$HATS(w) = HATS_0(w) + 2 \cdot \sum_{k=1}^{D} HATS_k(w) \tag{84}$$

H indexes:

$$H_k(w) = \sum_{i=1}^{n_A-1} \sum\nolimits_{j>i} h_{ij} \cdot w_i \cdot w_j \cdot \delta(d_{ij}; h_{ij}; k) \quad k = 0,1,2,\dots,D \tag{85}$$

H total index:

$$HT(w) = H_0(w) + 2 \cdot \sum_{k=1}^{D} H_k(w) \tag{86}$$

R indexes:

$$R_k(w) = \sum_{i=1}^{n_A-1} \sum\nolimits_{j>i} \frac{\sqrt{h_{ii} \cdot h_{jj}}}{r_{ij}} \cdot w_i \cdot w_j \cdot \delta(d_{ij}; k) \quad k = 1,2,\dots,D \tag{87}$$

R total index:

$$RT(w) = 2 \cdot \sum_{k=1}^{D} R_k(w) \tag{88}$$

Maximal R indexes:

$$R_k^+(w) = max_{ij}\left(\frac{\sqrt{h_i \cdot h_j}}{r_{ij}} \cdot w_i \cdot w_j \cdot \delta(d_{ij}; k)\right) \quad i \neq j \quad k = 1,2,\dots,D \tag{89}$$

Maximal R total index:

$$RT^+(w) = max_k(R_k^+(w)) \tag{90}$$

Here, n_A is the number of molecule atoms (hydrogen included), D is the topological diameter; d_{ij} is the topological distance between atoms i and j, and w_i is a physico-chemical property of the ith atom.

4.6. WHIM Descriptors

WHIM stands for Weighted holistic invariant molecular descriptors. They are based on statistical indexes calculated by projecting atoms along principal axes. WHIM descriptors capture 3D information regarding molecular size, shape, symmetry, and atom distribution with respect to invariant reference frames. They are calculated by calculating eigenvalues and eigenvectors of a weighted covariance matrix of the centered cartesian coordinates of the atoms of a molecule. This weighted covariance matrix is given by [1,3]

$$s_{jk} = \frac{\sum_{i=1}^{n_A} w_i \left(q_{ij} - \bar{q}_j\right)\left(q_{ik} - \bar{q}_k\right)}{\sum_{i=1}^{An_A} w_i} \tag{91}$$

where s_{jk} is the weighted covariance between the jth and kth atomic coordinates, n_A is the number of atoms, w_i is the weight of the ith atom, q_{ij} and q_{ik} represent the jth and kth coordinate $(j,\ k\ = x, y, z)$ of the ith atom, respectively, and $\bar{q}$ the corresponding average value.

Using this weighted covariance matrix, the following WHIM descriptors are calculated [1,3]:

d-WSIZ indexes:

$$\lambda_m \ \text{ for } m = 1,2,3 \tag{92}$$

WSIZ index:

$$T = \lambda_1 + \lambda_2 + \lambda_3 \tag{93}$$

WSIZ index:

$$A = \lambda_1\lambda_2 + \lambda_1\lambda_3 + \lambda_2\lambda_3 \tag{94}$$

WSIZ index:

$$V = \prod_{m=1}^{3}(1 + \lambda_m) - 1 = \mathrm{T} + \mathrm{A} + \lambda_1\lambda_2\lambda_3 \tag{95}$$

d-WSHA indexes:

$$\vartheta_m = \frac{\lambda_m}{\sum m^{\lambda} m} \quad \text{ for } m = 1,2,3 \tag{96}$$

WSHA index:

$$K = \frac{3}{4} \cdot \sum_{m=1}^{3} \left| \frac{\lambda_m}{\sum m^{\lambda} m} - \frac{1}{3} \right| \tag{97}$$

d-WDEN indexes:

$$\eta_m = \frac{\lambda_m^2 \cdot A}{\sum_i t_i^4} \quad \text{ for } m = 1,2,3 \tag{98}$$

WDEN index:

$$D = \eta_1 + \eta_2 + \eta_3 \tag{99}$$

d-WSYM indexes:

$$\gamma_m = \left\{1 - \left[\frac{n_s}{A} \cdot log_2 \frac{n_s}{A} + n_a \cdot \left(\frac{1}{A} \cdot log_2 \frac{1}{A}\right)\right]\right\}^{-1} \quad \text{ for } m = 1,2,3 \tag{100}$$

WSYM index:

$$G = (\gamma_1 \cdot \gamma_2 \cdot \gamma_3)^{1/3} \quad (101)$$

Here, λ refers to eigenvalues of the weighted covariance matrix; t refers to atomic coordinates with respect to the principal axes; n_A is the number of molecule atoms; n_s is the number of symmetric atoms along a principal axis; and n_a is the number of asymmetric atoms.

4.7. RDF Descriptors

Radial distribution function (RDF) descriptors were proposed based on an RDF that is quite often used for the interpretation of the diffraction patterns obtained in powder X-ray diffraction experiments. The RDF of an ensemble of n_A atoms can be interpreted as the probability distribution to find an atom in a spherical volume of radius R. The general form of the radial distribution function is represented [1,3] by

$$g(R) = f \cdot \sum_{i=1}^{n_A-1} \sum_{j=i+1}^{n_A} w_i \cdot w_j \cdot e^{-\beta \cdot (R - r_{ij})^2} \quad (102)$$

where f is a scaling factor, w are characteristic atomic properties of the atoms i and j, r_{ij} is the interatomic distance between the ith and jth atom, R is the radius of the spherical volume, and n_A is the number of atoms. β is the smoothing parameter which defines the probability distribution of the individual distances. PyL3dMD uses a β equal to 100 $Å^{-2}$. PyL3dMD calculates RDF vector of 30 values starting from 1.0 Å up to 15.5 Å using a step size of 0.5 Å for R.

4.8. Autocorrelation Descriptors

Various autocorrelation-based descriptors are calculated as [1,3]

3D Moreau-Broto autocorrelation (ATS) descriptors:

$$\{\mathrm{ATS_0}, \mathrm{ATS_1}, \mathrm{ATS_2}, \ldots, \mathrm{ATS_D}\}_w \quad (103)$$

Here,

$$ATS_0 = \sum_{i=1}^{n_A} w_i^2 \quad (104)$$

$$ATS_k = \frac{1}{2} \cdot \sum_{i=1}^{n_A} \sum_{j=1}^{n_A} w_i \cdot w_j \cdot \delta(r_{ij}; k) \quad (105)$$

Average ATS descriptors:

$$\overline{ATS_k} = \frac{1}{2\Delta_k} \cdot \sum_{i=1}^{n_A} \sum_{j=1}^{n_A} w_i \cdot w_j \cdot \delta(r_{ij}; k) \quad (106)$$

3D Geary (GATS) autocorrelation indices:

$$C_k = \frac{\frac{1}{2\Delta_k} \cdot \sum_{i=1}^{n_A} \sum_{j=1}^{n_A} (w_i - w_j)^2 \cdot \delta(r_{ij}; k)}{\frac{1}{(n_A - 1)} \cdot \sum_{j=1}^{n_A} (w_i - \overline{w})^2} \quad (107)$$

3D Moran (MATS) autocorrelation descriptors:

$$C_k = \frac{\frac{1}{\Delta_k} \cdot \sum_{i=1}^{n_A} \sum_{j=1}^{n_A} [(w_i - \overline{w}) \cdot (w_j - \overline{w})] \cdot \delta(r_{ij}; k)}{\frac{1}{n_A} \cdot \sum_{j=1}^{n_A} (w_i - \overline{w})^2} \quad (108)$$

PyL3dMD calculates ATS, GATS, and MATS descriptors vector of 30 values starting from 1.0 Å up to 15.5 Å using a step size of 0.5 Å for interatomic distance r_{ij}.

4.9. 3D-MoRSE Descriptors

3D-MoRSE stands for 3D-molecule representation of structures based on electron diffraction [15]. The 3D-MoRSE descriptors translate the 3D coordinates into a molecular code with a modified equation used in electron diffraction studies for preparing theoretical scattering curves [16,17].

$$\text{MoRSE} = \begin{cases} \sum_{i=1}^{n_A - 1} \sum_{j=i+1}^{n_A} w_i \cdot w_j & \text{if } s = 1 \\ \sum_{i=1}^{n_A - 1} \sum_{j=i+1}^{n_A} w_i \cdot w_j \cdot \frac{\sin[(s-1) \cdot r_{ij}]}{(s-1) \cdot r_{ij}} & \text{otherwise} \end{cases} \quad (109)$$

Here, n_A is the number of atoms, w is one of the atomic properties used as the atomic weightings, s is the scattering angle, and r_{ij} is the geometrical distance between the atoms i and j.

It is calculated for 30 evenly distributed values of s in the range of 0.5-15.0 $Å^{-1}$ from the 3D coordinates of the molecule. More details, including physical significance, of 3D-MoRSE descriptors can be found here [9]. These descriptors are usually calculated for molecules having 3D coordinates for all atoms, including hydrogen atoms.

Bibliography

[1] Todeschini, R., and Consonni, V., 2010, *Molecular Descriptors for Chemoinformatics*.

[2] Cao, D. S., Xu, Q. S., Hu, Q. N., and Liang, Y. Z., 2013, "ChemoPy: Freely Available Python Package for Computational Biology and Chemoinformatics," Bioinformatics, **29**(8), pp. 1092–1094.

[3] JERZY LESZCZYNSKI, 2010, *Recent Advances in QSAR Studies*.

[4] Consonni, V., Todeschini, R., and Pavan, M., 2002, "Structure/Response Correlations and Similarity/Diversity Analysis by GETAWAY Descriptors. 1. Theory of the Novel 3D Molecular Descriptors," J Chem Inf Comput Sci, **42**(3), pp. 682–692.

[5] Consonni, V., Todeschini, R., Pavan, M., and Gramatica, P., 2002, "Structure/Response Correlations and Similarity/Diversity Analysis by GETAWAY Descriptors. 2. Application of the Novel 3D Molecular Descriptors to QSAR/QSPR Studies," J Chem Inf Comput Sci, **42**(3), pp. 693–705.

[6] Todeschini, R., and Gramatica, P., 1998, "New 3D Molecular Descriptors: The WHIM Theory and QSAR Applications," Perspectives in Drug Discovery and Design, **9--11**, pp. 355–380.

[7] Todeschini, R., and Consonni, V., 2000, *Handbook of Molecular Descriptors*.

[8] Todeschini, R., and Gramatica, P., 1997, "The Whim Theory: New 3D Molecular Descriptors for Qsar in Environmental Modelling," SAR QSAR Environ Res, **7**(1–4), pp. 89–115.

[9] Devinyak, O., Havrylyuk, D., and Lesyk, R., 2014, "3D-MoRSE Descriptors Explained," J Mol Graph Model, **54**, pp. 194–203.

[10] Mattice, W. L., and Suter, U. W., 1994, *Conformational Theory of Large Molecules*, Wiley-Interscience, New York.

[11] Theodorou, D. N., and Suter, U. W., 1985, "Shape of Unperturbed Linear Polymers: Polypropylene," Macromolecules, **18**(6), pp. 1206–1214.

[12] Flory, Paul. J., and Volkenstein, M., 1969, "Statistical Mechanics of Chain Molecules," Biopolymers, **8**(5), pp. 699–700.

[13] Sauer, W. H. B., and Schwarz, M. K., 2003, "Molecular Shape Diversity of Combinatorial Libraries: A Prerequisite for Broad Bioactivity.," ChemInform, **34**(36).

[14] Firth, N. C., Brown, N., and Blagg, J., 2012, "Plane of Best Fit: A Novel Method to Characterize the Three-Dimensionality of Molecules," J Chem Inf Model, **52**(10), pp. 2516–2525.

[15] Schuur, J. H., Selzer, P., and Gasteiger, J., 1996, "The Coding of the Three-Dimensional Structure of Molecules by Molecular Transforms and Its Application to Structure-Spectra Correlations and Studies of Biological Activity," J Chem Inf Comput Sci, **36**(2), pp. 334–344.

[16] Soltzberg, L. J., and Wilkins, C. L., 1977, "Molecular Transforms: A Potential Tool for Structure-Activity Studies," J Am Chem Soc, **99**(2), pp. 439–443.

[17] Mauri, A., 2020, "AlvaDesc: A Tool to Calculate and Analyze Molecular Descriptors and Fingerprints," Ecotoxicological QSARs, pp. 801–820.
